# The negative elongation factor NELF promotes induced transcriptional response of *Drosophila* ecdysone-dependent genes

**DOI:** 10.1038/s41598-020-80650-1

**Published:** 2021-01-08

**Authors:** Marina Yu. Mazina, Elena V. Kovalenko, Nadezhda E. Vorobyeva

**Affiliations:** Institute of Gene Biology, Russian Academy of Sciences, Moscow, 119334 Russia

**Keywords:** Gene regulation, Epigenetics, Transcription

## Abstract

For many years it was believed that promoter-proximal RNA-polymerase II (Pol II) pausing manages the transcription of genes in *Drosophila* development by controlling spatiotemporal properties of their activation and repression. But the exact proteins that cooperate to stall Pol II in promoter-proximal regions of developmental genes are still largely unknown. The current work describes the molecular mechanism employed by the Negative ELongation Factor (NELF) to control the Pol II pause at genes whose transcription is induced by 20-hydroxyecdysone (20E). According to our data, the NELF complex is recruited to the promoters and enhancers of 20E-dependent genes. Its presence at the regulatory sites of 20E-dependent genes correlates with observed interaction between the NELF-A subunit and the ecdysone receptor (EcR). The complete NELF complex is formed at the 20E-dependent promoters and participates in both their induced transcriptional response and maintenance of the uninduced state to keep them ready for the forthcoming transcription. NELF depletion causes a significant decrease in transcription induced by 20E, which is associated with the disruption of Pol II elongation complexes. A considerable reduction in the promoter-bound level of the Spt5 subunit of transcription elongation factor DSIF was observed at the 20E-dependent genes upon NELF depletion. We presume that an important function of NELF is to participate in stabilizing the Pol II-DSIF complex, resulting in a significant impact on transcription of its target genes. In order to directly link NELF to regulation of 20E-dependent genes in development, we show the presence of NELF at the promoters of 20E-dependent genes during their active transcription in both embryogenesis and metamorphosis. We also demonstrate that 20E-dependent promoters, while temporarily inactive at the larval stage, preserve a Pol II paused state and bind NELF complex.

## Introduction

Over the years, researchers have aimed to discover and characterize proteins and protein complexes that regulate the transcription of genes. To date, multiple complexes have been described, and we are now entering a breathtaking new phase of research that allows us to find a suitable place for each regulator in the complex multi-step process of eukaryotic transcription. A detailed system of model genes is required for the experimental modeling of the transcriptional process. For this purpose, we chose a set of developmental genes that can be rapidly induced by the addition of 20-hydroxyecdysone (20E) hormone in *Drosophila* cell culture^1^. In our previous works we have described in detail the primary stages of 20E-dependent induction and found new molecular partners of the EcR/Usp ecdysone sensor^2,3^. The present study aims to characterize the molecular complex responsible for the organization of the Pol II pause at 20E-dependent genes.

The phenomenon of promoter-proximal Pol II pausing (Pol II pausing) was first described in a study of *Drosophila* heat-shock protein genes^4^. RNA polymerase II was shown to be recruited at the promoters of these genes in their inactive state, and stress-induced activation of the heat shock genes resulted in Pol II release from their promoters into the gene bodies. Subsequent research has proven that the Pol II pause is an important regulatory phenomenon for many genes in various organisms^5–7^.

Early research indicated that some genes regulated by 20E use Pol II pausing as a mechanism for controlling their transcription^8,9^. However, the mechanism guiding Pol II pausing at 20E-dependent promoters was so far not described. Previously, we observed the presence of Pol II on many 20E-dependent promoters in their inactive state, and showed that the induction of 20E-dependent transcription leads to a change in the degree of Pol II phosphorylation rather than an increase in the amount of Pol II associated with the promoter^2^. In other words, we showed that 20E-dependent genes are regulated via Pol II pausing. In a recent work we revealed an interaction between the EcR/Usp sensor and the NELF-A subunit of the Negative elongation factor (NELF) and hypothesized that the latter may be responsible for Pol II pausing at 20E-dependent promoters^3^.

NELF is the most well-known factor involved in Pol II pausing at eukaryotic genes, where it cooperates with the DSIF complex to halt Pol II elongation^10^. The NELF- and DSIF-mediated transcription block is usually overcome by p-TEF-dependent phosphorylation, which is stimulated by transcriptional activators^11^. The NELF complex consists of four protein subunits that are evolutionarily conserved and are inherent in organisms that regulate transcription by Pol II pausing^12^. Currently, the described molecular functions of NELF are not limited to establishing a Pol II paused state^13^, and NELF was even found to be recruited to the promoters of actively transcribed genes^9,14^. The exact functions of NELF at the promoters of active genes are currently only poorly understood, but there is evidence for its role in mRNA processing and loading of the Cap-associated complex^15,16^ as demonstrated by the interaction between the NELF-E subunit and RNA in the Pol II paused complex^17^.

Pol II pausing is involved in the dynamic transcriptional regulation of developmental genes^18^. Namely, Pol II paused promoters participate in organization of 3D chromatin topology in embryogenesis by anchoring contacts coming from developmental enhancers^19^. It is believed that the main role of the Pol II pause in development is to synchronize transcriptional activation in individual cells of the embryo^20,21^. It was shown that the Pol II pause-derived temporal synchronization of gene activation is crucial for the coordination of cellular behavior at the most important stages of morphogenesis (e.g. during gastrulation)^20^. However, there is little information regarding the factors responsible for maintenance of the Pol II pause during the early hours of development. Our present study brings forth new insights by describing the role of NELF in transcriptional regulation of one of the central groups of genes in *Drosophila* development whose transcription is induced by 20E hormone.

Recently, we identified novel protein interactors of the EcR/Usp heterodimer using in vivo proximity-dependent biotin ligation^2^, including the NELF-A subunit. We validated this interaction in a previous report by co-immunoprecipitation of these proteins from the prepupal nuclear extract (where the ecdysone cascade is normally activated). We also observed a decrease in the 20E-dependent transcriptional induction of some genes upon NELF-A knockdown. Here, we describe the relationships between EcR and the NELF complex in detail and propose a mechanism for NELF function at 20E-dependent genes.

## Materials and methods

### Treatment of *Drosophila* S2 cells

*Drosophila* Schneider cell line 2 (S2) cells were maintained at 25 °C in ecdysone-free Schneider’s insect medium (Sigma) containing 10% FBS (HyClone). The treatment of S2 cells with 20-hydroxyecdysone (20E) (H5142, Sigma-Aldrich) was performed at a final concentration of 0.3 μM, as was described previously^1,2^. As different studies have employed various duration of 20E treatment to induce transcription, we limited our approach to only identify direct targets of 20E by limiting the time of 20E treatment to 1 h. This prevents primary-induced transcripts (which are in turn transcription factors) to be translated and shuttled back to the nucleus to affect transcription of their targets.

Immunoprecipitation^22^ and RNA interference-mediated knockdowns of GFP, NELF-A, B, D and E subunits of NELF complex, EcR and Spt5 in *Drosophila* S2 cells^2^ were performed as previously described. Efficiency of RNA knockdown was evaluated by qPCR and Western-blot and provided at Fig. S1. Chemo-luminescence signal was detected under ChemiDoc gel imager (Bio-Rad) and photographed using Image lab 4.1 software. All full-size images of Western blots are provided in the Supplementary information file. We regret not being able to provide the visible bottom edges of some full-size Western blots. They were lost during photographing to ensure the best possible resolution for meaningful lines.

### ChIP, ChIP-Seq

The chromatin immunoprecipitation (ChIP) was performed exactly as previously described^23^. ChIP-Seq libraries were obtained using the NEBNext DNA library preparation kit (New England Biolabs). Only the library fragments of 250–500 bp were subjected to NGS sequencing. New generation sequencing was performed by Evrogen (evrogen.ru) with the Illumina NovaSeq6000 sequencer. For each of the ChIP‐Seq libraries, approximately 5–14 millions of unique paired mappable reads were obtained. The paired‐end reads in FastQ format were mapped to the *Drosophila* genome assembly dm6 using Bowtie2^24^ and filtered (with minimum MAPQ quality score = 5).

BigWig files were generated using bamCoverage 3.0.2 with scores representing number of reads normalized by the size of the library (the protein binding levels were normalized to the genome content—calculated as RPGC: number of reads per bin/(total number of mapped reads * fragment length/effective genome size)^25^. The final bigwig files (representing the protein binding profiles) were obtained using bigwigcompare tool as ratio of ChIP signal to Input (all inputs were preliminary smoothed over a 1 kb window). Pile-up profiles were calculated as a median level of protein binding for the distributions of Rpb3, NELF-A, NELF-B and NELF-E near TSSs and Rpb3, NELF-A, NELF-B, NELF-E and CBP/Neijre near enhancers of 20E-dependent genes in *Drosophila* S2 cells. Pile-up profiles for the distributions of Rpb3, NELF-A and Pol II S2P in different stages of *Drosophila* development were also calculated as a median level. The CBP/Neijre binding sites in *Drosophila* S2 cells were defined by MACS2 with the following parameters: -gsize ‘120,000,000’ -keep-dup ‘1’-qvalue ‘0.01’ -mfold ‘5’ ‘30’ –bw ‘375’ 2 >  and 1 > macs2_stderr^26^. Corresponding input DNA was used as a control for peak calling.

**Figure 1:**
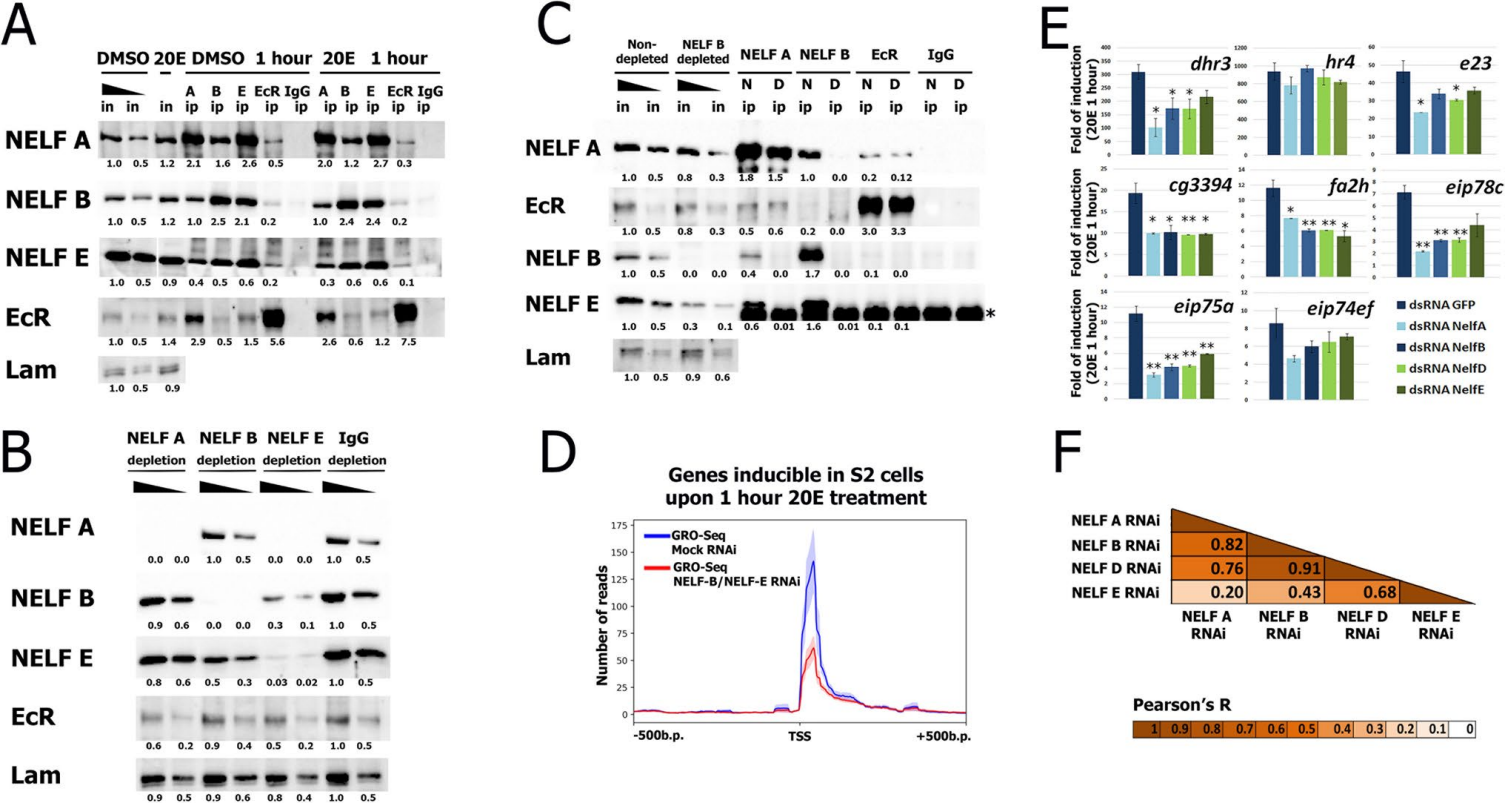
NELF complex interacts with EcR receptor and affects transcription of 20E-dependent genes. (**A**) Immunoprecipitations (IPs) from nuclear protein extracts of 1-h 20E- and DMSO- treated *Drosophila* S2 cells. Immunoprecipitations were performed with antibodies against NELF-A, NELF-B, NELF-E and EcR (a serum of non-immunized rabbits (IgG ip) was used as a negative control), which is indicated on the top of the figure. Western blots were stained with the corresponding antibodies indicated on the left of the figure. Anti-lamin staining was used as loading control. All input and IP samples were loaded on a single western blot. Western blot stained with anti-NELF-E antibodies included additional titration line between the control and treated cells inputs, which was cut for the uniform presentation of the results. The DMSO-treated input was loaded in the serial loadings (1, 0.5) and used as the calibration for measuring the intensities of the IP lanes in a TotalLab program. The numbers below the IPs represent the portions of precipitated proteins relative to loaded DMSO-treated input. The full-size original Western blots are provided in Fig. S11. (**B**) Depletion of NELF subunits from the protein nuclear extract of uninduced *Drosophila* S2 cells. Depletions were performed with antibodies against NELF-A, NELF-B and NELF-E (a serum of non-immunized rabbits (IgG) was used as in a negative depletion control), which is indicated on the top of the figure. Western blots were stained with the corresponding antibodies indicated on the left of the figure. Anti-lamin staining was used as loading control. All input and depletion samples were loaded on a single western blot. All the depletions were loaded in the same serial loadings (1, 0.5). IgG depletion was used as the calibration for measuring the intensities of the rest of the depletions in a TotalLab program. The numbers below the depletions represent the portions of loaded proteins relative to loaded IgG depletion. The full-size original Western blots are provided in Fig. S12. (**C**) Immunoprecipitations (IPs) from nuclear protein extracts of uninduced *Drosophila* S2 cells, with NELF-B depletion (D—depleted) or without it (N—non-depleted). Immunoprecipitations were performed with antibodies against NELF-A, NELF-B, NELF-E and EcR (a serum of non-immunized rabbits (IgG ip) was used as a negative control), which is indicated on the top of the figure. Western blots were stained with the corresponding antibodies indicated on the left of the figure. Anti-lamin staining was used as loading control. All input and IP samples were loaded on a single western blot. The inputs were loaded in the serial loadings (1, 0.5) and the non-depleted input was used as the calibration for measuring the intensities of the NELF-B depleted input and IP lanes in a TotalLab program. The numbers below the NELF-B depleted input and IPs represent the portions of precipitated proteins relative to loaded non-depleted input. The full-size original Western blots are provided in Fig. S13. (**D**) The level of 5′proximal transcription at 20E-dependent promoters in *Drosophila* S2 cells concomitantly treated with dsRNAs against NELF-B and NELF-E or dsRNA to beta-galactosidase (as mock RNAi) was estimated by GRO-Seq (the GRO-Seq data were taken from GSM3274627 and GSM3274628 (described for the first time in ref.^29^) and the list of 20E-dependent genes from our previous study^1^). Pile-up profiles were calculated as a mean of a GRO-Seq signal. The Y-axis units represent number of reads. The standard error is displayed on the graphs as lighter area around the main line of the profile. (**E**) Transcriptional induction levels of 20E-dependent genes in 1-h 20E-induced *Drosophila* S2 cells relative to DMSO-treated. The impact of NELF-A, NELF-B, NELF-D and NELF-E subunits of NELF complex on 20E response was estimated using RNA interference-mediated knockdown with corresponding dsRNA (GFP dsRNA was taken as a mock RNAi). Transcriptional levels were assessed by qRT-PCR. The Y-axis units represent fold of transcription induction which was calculated as a ratio between transcriptional level of corresponding gene in a particular experiment and mock RNAi treated uninduced cells sample (and normalized on tubulin mRNA). The data are mean values from three independent experiments, error bars represent standard deviations. Asterisks indicate significance levels (Student’s t-test), **—*p*-value < 0.01, *—*p*-value < 0.05. Statistical analysis was performed relative to the transcriptional induction levels of the genes in GFP dsRNA treated S2 cells. (**F**) Pearson’s correlation matrix for the impact of NELF-A, NELF-B, NELF-D and NELF-E subunits knockdowns on transcriptional induction levels of 20E-dependent genes in 1-h 20E-induced *Drosophila* S2 cells relative to DMSO-treated (the list of the genes is the same as on **E**). The heat map reflects the indicated R values.

**Figure 2:**
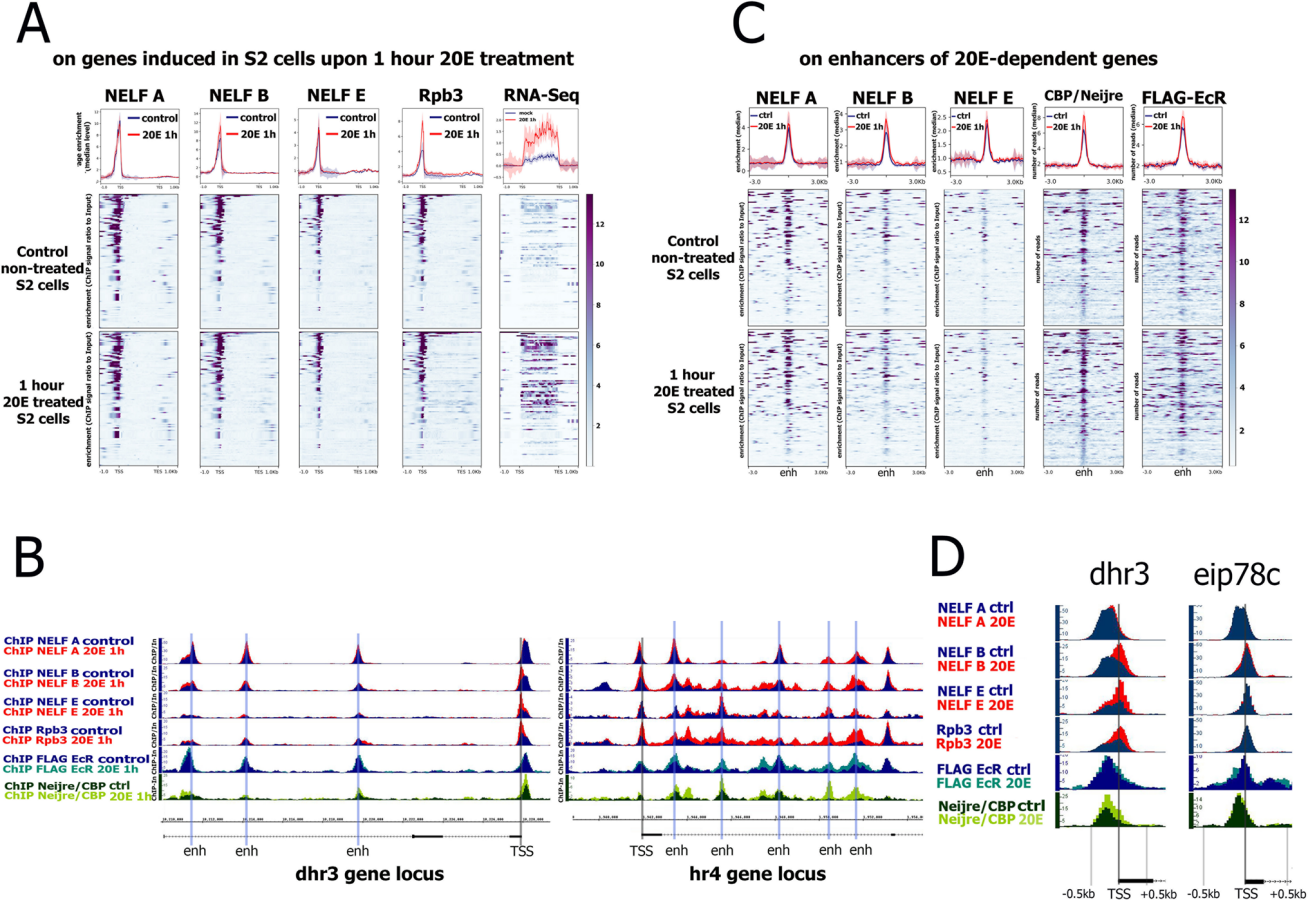
NELF complex subunits colocalize with promoters and enhancers of 20E-dependent genes. (**A**) Average distribution of NELF-A, NELF-B, NELF-E (NELF subunits) and Rpb3 (Pol II subunit) binding and an average of RNA-seq signal on 20E -dependent genes upon 1 h 20E treatment (20E 1 h) and in DMSO-treated (Control) *Drosophila* S2 cells. The list of 20E-dependent genes induced in *Drosophila* S2 cells upon 1 h 20E treatment was estimated in a previous study^1^. NELF-A, NELF-B, NELF-E and Rpb3 binding levels were calculated as an enrichment (ratio of corresponding ChIP-Seq signal over input DNA). Average profiles were generated using metagene mode (introns were ignored) and calculated as a median of NELF-A, NELF-B, NELF-E and Rpb3 binding level and RNA-seq signal, respectively. The standard error is displayed on the graphs as lighter area around the main line of the profiles. (**B**) Binding profiles of NELF-A, NELF-B, NELF-E, Rpb3, FLAG-EcR and CBP/Neijre at *dhr3*, *hr4* 20E-inducible genes. Grey lines indicate TSSs and enhancers (enh). Protein binding levels were estimated by ChIP-Seq. ChIP-Seq experiments were performed using 1 h 20E- or DMSO-treated *Drosophila* S2 cells (which is indicated on the left of the figure as 20E 1 h and Control, respectively). ChIP-Seq data on FLAG-EcR binding were loaded from GSE139316 and described previously in ref.^3^. (**C**) Average distribution of NELF-A, NELF-B, NELF-E, CBP/Neijre (GSE156847) and FLAG-EcR (GSE139316) on enhancers of 20E-dependent genes upon 1 h 20E treatment (20E 1 h) and in DMSO-treated (Control) *Drosophila* S2 cells. NELF-A, NELF-B, NELF-E binding levels were calculated as an enrichment (ratio of corresponding ChIP-Seq signal over input DNA). CBP/Neijre and FLAG-EcR binding levels were calculated as difference between ChIP-Seq signal and Input. Pile-up profiles were calculated as a median of protein binding levels. The standard error is displayed on the graphs as lighter area around the main line of the profiles. (**D**) Binding profiles of NELF-A, NELF-B, NELF-E, Rpb3, FLAG-EcR and CBP/Neijre at TSSs of *dhr3 and eip78c* 20E-inducible genes. Protein binding levels were estimated by ChIP-Seq. ChIP-Seq experiments were performed using 1 h 20E- or DMSO-treated *Drosophila* S2 cells (which is indicated on the left of the figure as 20E 1 h and Control, respectively). ChIP-Seq data on FLAG-EcR binding were obtained previously^3^.

**Figure 3:**
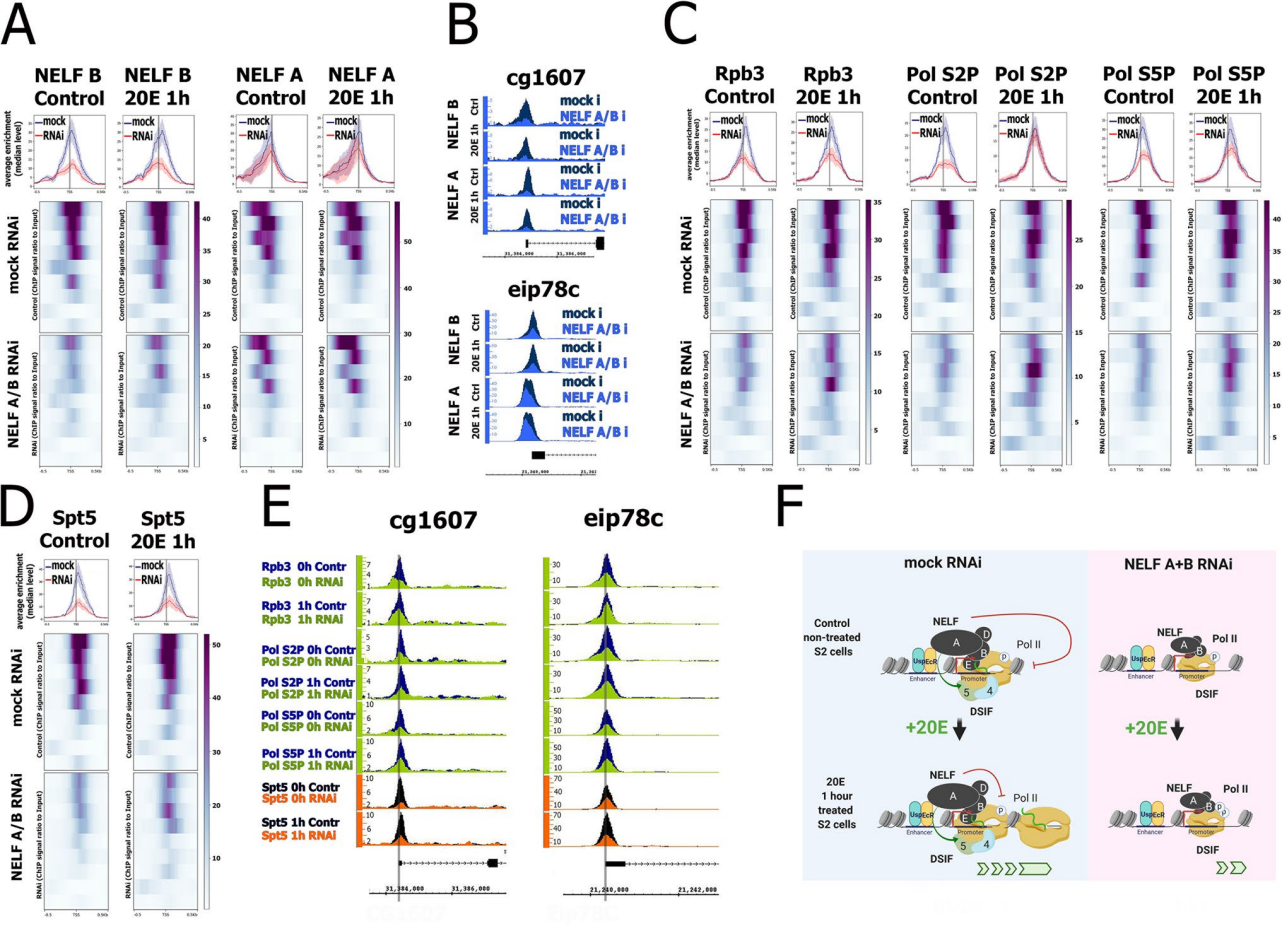
Depletion of NELF affects promoter-bound level of RNA polymerase II and DSIF in both the induced and uninduced states of 20E-dependent genes. (**A**) Average distribution of NELF-B and NELF-A subunits binding in 1 h 20E treated (20E 1 h) or in DMSO-treated (control) *Drosophila* S2 cells concomitantly treated with dsRNAs to NELF-B and NELF-A (RNAi) or dsRNA corresponding to GFP transcript (mock). Pile-up profiles were generated for the TSSs of nine 20E-dependent genes whose promoter regions demonstrated more than twofold decrease in NELF-B binding level upon treatment with dsRNA against NELF-A/NELF-B. All profiles were calculated as a median of proteins binding levels. The standard error is displayed on the graphs as lighter area around the main line of the profiles. (**B**) Binding profiles of NELF-B and NELF-A on TSS of *cg1607* and *eip78c* 20E-dependent genes in 1 h 20E treated (20E 1 h) or in DMSO-treated (control) *Drosophila* S2 cells concomitantly treated with dsRNAs to NELF-B and NELF-A (RNAi) or dsRNA corresponding to GFP transcript (mock). Protein binding levels were estimated by ChIP-Seq and represent the enrichment of ChIP-Seq signal over input. (**C**,** D**) Average binding distribution of Rbp3, Pol II, phosphorylated on Ser2 of CTD (Pol S2P), Pol II, phosphorylated on Ser5 of CTD (Pol S5P) (**C**) and Spt5 (**D**) in 1 h 20E treated (20E 1 h) or in DMSO-treated (control) *Drosophila* S2 cells concomitantly treated with dsRNAs to NELF-B and NELF-A (RNAi) or dsRNA corresponding to GFP transcript (mock). Pile-up profiles were generated for the TSSs of nine 20E-dependent genes whose promoter regions demonstrated more than twofold decrease in NELF-B binding level upon treatment with dsRNA against NELF-A/NELF-B. All profiles were calculated as a median of proteins binding level which was estimated as an enrichment. The standard error is displayed on the graphs as lighter area around the main line of the profiles. (**E**) Binding profiles of Rbp3, Pol II, phosphorylated on Ser2 of CTD (Pol S2P), Pol II, phosphorylated on Ser5 of CTD (Pol S5P) and Spt5 on *cg1607* and *eip78c* 20E-inducible promoters. Protein binding levels were estimated by ChIP-Seq as an enrichment. ChIP-Seq experiments were performed using *Drosophila* S2 cells upon 1 h 20E induction (20E 1 h) or DMSO-treated cells (control) concomitantly treated with dsRNAs against NELF-B and NELF-A (RNAi) or dsRNA corresponding to GFP transcript (mock). (**F**) Proposed model describing a role of NELF complex in transcriptional regulation of an average 20E-dependent gene (image created using BioRender.com). NELF complex is recruited to 20E-dependent promoters, possibly through interaction of NELF-A subunit with EcR/Usp dimer at 20E-dependent enhancers. NELF organizes Pol II pause on uninduced 20E-dependent genes and its depletion causes the decrease in Pol II promoter-bound level. During induced transcription NELF promotes formation of Pol II-elongating complex by stabilizing the complex between Pol II and DSIF.

**Figure 4:**
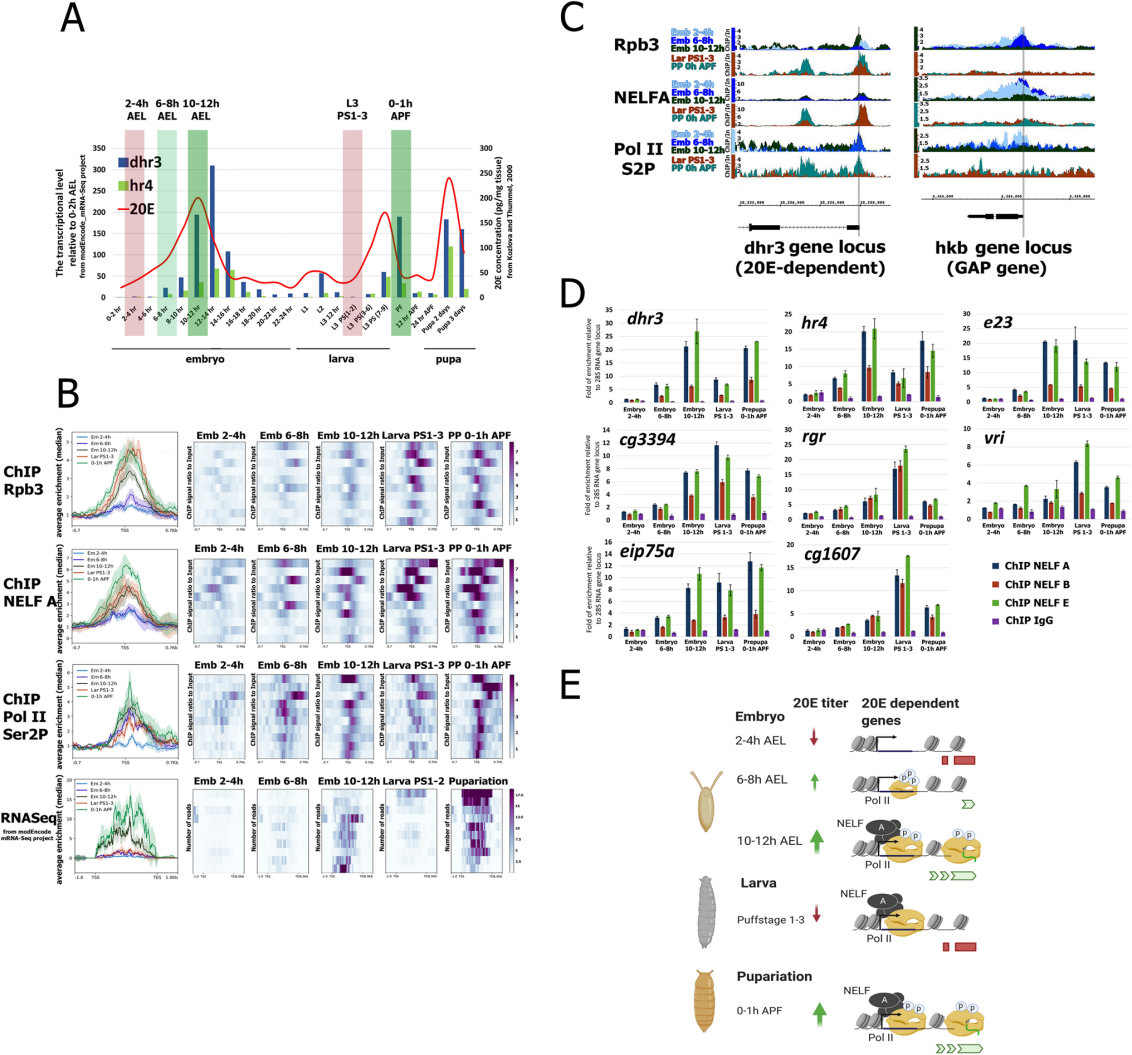
NELF organizes Pol II pause on 20E-dependent promoters during larva-to-pupa transition. (**A**) The transcription levels of *dhr3* (blue columns) and *hr4* (green columns) 20E-dependent genes during ontogenesis of *Drosophila* (from modENCODE_mRNA-Seq data^30^) relative to their transcription during 0–2 h after egg laying (AEL) (Y axis on the left of the figure). Purple and green transparent lines indicate the investigated developmental stages (2–4 h AEL, 6–8 h AEL, 10–12 h AEL, 3^rd^ stage larvae 12 h before pupariation (L3 PS1-3), 0–1 h after puparium formation (APF)). Red line indicates an average 20E concentration in the tissues (Y axis on the right) during *Drosophila* development (according to ref.^35^). (**B**) Average distribution of Rbp3, NELF-A and Pol II Ser2P binding on stably induced 20E-dependent genes at different developmental stages. A set of stably induced 20E-dependent genes was selected from the list of genes induced in *Drosophila* S2 cells after 1-h treatment with 20E, based on a criterion—to demonstrate an increase in the transcriptional level both by 10–12 h AEL and during puparium formation (according to modENCODE_mRNA-Seq data^30^). Protein binding levels were estimated by ChIP-Seq from corresponding developmental stage and calculated as an enrichment. Averaged RNA-seq profiles were calculated for 20E-dependent genes on different developmental stages, using the data from modENCODE_mRNA-Seq project^30^. Pile-up profiles were calculated as a median of Rbp3, NELF-A and Pol II Ser2P binding levels. The standard error is displayed on the graphs as lighter area around the main line of the profiles. (**C**) Binding profiles of Rpb3, NELF-A and Pol II S2P on *dhr3* 20E-inducible locus and on *hkb* locus (GAP gene) during *Drosophila* development. Protein binding levels were estimated by ChIP-Seq from corresponding developmental stage and calculated as an enrichment. Grey lines indicate the promoters. (**D**) ChIP analysis of NELF-A, NELF-B and NELF-E binding to promoters of some stably induced 20E-dependent genes at different developmental stages of *Drosophila*. The nuclear chromatin extracts were obtained from the same developmental stages as at (**B**, **C**) (indicated on the bottom of the histograms). Relative binding levels of NELF subunits were assessed by qRT-PCR. The Y-axis units represent the fold of enrichment relative to the control region (28S ribosomal RNA gene locus). ChIP with a serum of non-immunized rabbits (IgG) was used to assess the specificity of antibodies against NELF subunits. The data are mean values from three independent experiments, error bars represent standard deviations. (**E**) Proposed model of the changes in the state of 20E-stably induced promoters during *Drosophila* development (image created using BioRender.com).

A list of 20E-dependent genes (induced in S2 Schneider cells upon 1 h 20E treatment) was obtained previously^1^. In the present study (Figs. 1, 2) we analyzed only the genes whose transcription demonstrated more than twofold induction upon 20E treatment (this exact list is provided in Table S1 in a .bed format). For the analysis, provided in Fig. 3, we selected only 9 20E-dependent transcripts whose promoter regions demonstrated more than twofold decrease in NELF B binding upon treatment with dsRNA against NELF-A/NELF-B (these top9 transcripts are listed in Table S2 as a .bed). The remaining 27 transcripts of 20E dependent genes which NELF-B promoter-bound level doesn’t change upon NELF-A/NELF-B RNAi were used for the Fig. S2 and listed in Table S3 as a .bed. For the analysis in Fig. 4 we selected only 20E-dependent genes demonstrating transcriptional induction both at 10–12 h of embryogenesis and during puparium formation according to the study provided by Graveley et al^27^. A list of 20E-dependent genes (12 transcripts), expressed both during embryogenesis and pupariation, is provided in Table S3.

A list of enhancers of rapidly induced 20E-dependent genes was generated based on the following criteria: presence of a CBP/Neijre peak, absence of a TSS, and location within a 20E-induced gene (a list of 20E-induced genes was taken from Table S1).

The Galaxy-P platform was used for analysis of ChIP-Seq data^28^. All obtained ChIP-Seq data were deposited into the Gene Expression Omnibus—GSE156847.

GRO-Seq data were described previously and deposited as GSM3274627 and GSM3274628^29^. ChIP-Seqs of FLAG-EcR from S2 Schneider cells were deposited and described previously as GSE139316^3^. RNA-Seqs from 1 h DMSO- or 20E-treated S2 Schneider cells were described previously but were not deposited in GEO (thus now we provide these data in GSE156847^1^. Developmental RNA-seq data were obtained from modENCODE_mRNA-Seq project^30^—downloaded from the http://data.modencode.org (described for the first time in ref.^27^). We did not use any figures or text from the previously published manuscripts—only data deposited in free access databases.

### Statistical analysis

Pile-up profiles for Fig. 1D were calculated using DeepTool2 as a mean of GRO-Seq signal (estimated as a number of reads at the corresponding position of the *Drosophila* genome)^25^. The standard error was calculated and displayed on the graphs as lighter areas around the main line of the profile.

Transcriptional induction level in Fig. 1E and in Fig. S5 was calculated as a ratio between transcriptional level of corresponding gene in a particular experiment and mock RNAi treated uninduced cells sample (and normalized on tubulin mRNA). The data are mean values from three independent biological experiments, error bars represent standard deviations. Asterisks indicate significance levels (Student’s t-test), **—*p*-value < 0.01, *—*p*-value < 0.05. Statistical analysis was performed relative to the transcriptional induction levels of the genes in GFP dsRNA treated S2 cells.

The correlation for the impact of NELF-A, NELF-B, NELF-D and NELF-E subunits knockdowns on transcriptional induction levels of 20E-dependent genes provided in Fig. 1F was calculated using Pearson’s correlation test. The heat map reflects the calculated R values.

Pile-up profiles of ChIP-Seq and RNA-Seq signals in Figs. 2, 3, 4, Figs. S5, S7 and Fig. S9 were calculated as a median using DeepTool2^25^. NELF-A, NELF-B, NELF-E, Rpb3, Pol II Ser2P and Pol II Ser5P binding levels were estimated as an enrichment (ratio of corresponding ChIP-Seq signal over input DNA). CBP/Neijre and FLAG-EcR binding levels were estimated as difference between ChIP-Seq signal and Input. RNA-Seq signal represent a number of reads at the corresponding position. The standard error is displayed on the graphs as lighter area around the main line of the profiles.

NELF-A, NELF-B and NELF-E binding levels at the Fig. 4D and NELF-B and EcR binding levels at the Fig. S8 were calculated as an enrichment of ChIP signal at the corresponding locus to the control region (28S ribosomal RNA gene locus). Mean levels were provided basing on results of three independent biological experiments. Standard deviations were calculated and provided as error bars.

Transcriptional levels in Fig. S1 represent transcriptional level of the subunit in the corresponding knockdown compared to subunit expression in K- in percent. Data are mean values from three independent experiments, error bars represent standard deviations.

### Collection of the material corresponding to different *Drosophila* developmental stage

The flies of Oregon-R-modENCODE stock were used (corresponds to Bloomington stock 25,211). Embryos were collected in the fly cages for 2 h using apple juice agar plates. Then plates were incubated at the 25 °C for the required period of time. The time after egg laying (AEL) was calculated starting from the moment the agar plates were placed in the fly cages.

L3 larvae, corresponding to the puff stage 1–3, were collected by culturing larvae in the fly media supplemented with 0.05% bromophenol blue to mark the guts of feeding animals (L3 larvae had dark blue gut). Prepupae corresponding to 0–1 h after puparium formation (APF) were collected according to description of this developmental stage: white motionless prepupae starting to evert their anterior spiracles.

For the ChIP-Seq experiments embryos were dechorionized, washed and homogenized in a buffer (60 mM KCl, 15 mM NaCl, 4 mM MgCl_2_, 15 mM HEPES pH7.6, 0,5% Triton X-100) containing 0.7% of formaldehyde for 10 min and incubated for 5 min with 125 mM Glycine. Then cells were washed for three times with homogenization buffer. The remaining ChIP protocol was performed as described previously^23^.

To validate our material collected from the various developmental stages we measured transcription of *dhr3* and *hr4* genes induced by 20E (these data are provided in Fig. S3).

### Antibodies

Pol II S2P and Pol II S5P antibodies were purchased from Sigma-Aldrich (Ab5095 and Ab5131). For the ChIP-Seq analysis of CBP/Neijre, Rpb3, Spt5, NELF-A and NELF-E we used previously described antibodies^2,3^. Antibodies against NELF-B (epitope corresponding to 150–594 amino acid residues) were generated in this study. The epitopes for antibody production were expressed as 6 × His-tagged fusion proteins in *Escherichia coli*, affinity-purified on Ni Sepharose 6 Fast Flow (GE Healthcare), according to the manufacturer’s protocol, and injected into rabbits, following the standard immunization procedure. Antibodies were affinity-purified using the same epitopes as were used for immunization. The specificity of antibodies against EcR, Spt5, NELF A and NELF B was characterized in the RNA interference experiments by the specific depletion of corresponding proteins (Figs. S1–S4). Antibodies production was performed according to procedures outlined in the NIH (USA) Guide for the Care and Use of Laboratory Animals. The protocol used was approved by the Committee on Bioethics of the Institute of Gene Biology of the Russian Academy of Sciences. All procedures were performed under conditions designed to minimize suffering.

## Results

### EcR and NELF subunits co-immunoprecipitate from nuclear extracts of 20E-induced and uninduced *Drosophila* S2 cells

Previously, *Drosophila* S2 cells were shown to be suitable for the investigation of the ecdysone response as most ecdysone cascade genes are expressed upon 20E treatment^1^. We used this system to evaluate the influence of the 20E hormone on the interaction between EcR and NELF. We first investigated interactions between NELF subunits and EcR in *Drosophila* S2 nuclear extracts using specific antibodies (Fig. 1A). We obtained specific antibodies against 3 out of 4 subunits of *Drosophila* NELF: NELF-A, NELF-B and NELF-E. Antibodies against NELF-A and NELF-E were previously briefly characterized^2,3^. In this report we provide their additional description together with information on properties of newly obtained antibodies against NELF-B (Fig. S1).

All antibodies against NELF subunits successfully co-immunoprecipitated EcR, with antibodies against NELF-A demonstrating a stronger interaction. This may suggest a closer connection between EcR and this particular subunit of NELF, which is additionally corroborated by proximity-dependent biotin labeling of NELF-A with EcR/Usp APEX/BioID fusions^3^. In a reciprocal experiment, antibodies against EcR precipitated NELF-B, NELF-E, and, with a bit higher strength, the NELF-A subunit. We have quantified the amount of precipitated proteins relative to loaded inputs to more clearly demonstrate the observed interactions. Interestingly, interactions between EcR and NELF subunits were found to be independent of 20E hormone in the medium, suggesting that NELF binds the main ecdysone sensor both in the 20E-induced and uninduced *Drosophila* S2 cells.

### NELF-A can interact with EcR as a part of a partial NELF subcomplex lacking NELF-B subunit

An observed stronger association of EcR with NELF-A subunit than with NELF-B and NELF-E proposes the possibility of NELF-A interacting with EcR separately from the complete NELF complex. It is well proven that all NELF factor subunits are involved in the interaction with Pol II complex, but the existence of its partial complexes during loading onto chromatin cannot be ruled out^17^. To study this possibility, we performed a depletion of different NELF subunits from the protein nuclear extract of uninduced *Drosophila* S2 cells using specific antibodies against NELF-A, NELF-B and NELF-E (Fig. 1B).

The depletion experiment indeed revealed the existence of some subcomplexes formed by NELF subunits. The NELF-B depletion almost did not affect the level of NELF-A subunit in the nuclear protein extract but it decreased the level of NELF-E at least two times. Contrariwise, NELF-A depletion did not decrease the level of NELF-B and NELF-E. NELF-E depletion was shown to completely withdraw NELF-A from the nuclear extract and decrease the level of NELF-B. Thus, we suggest that NELF-A subunit can exist in *Drosophila* nucleus in a state of a separate subcomplex, also containing NELF-E.

As NELF-A was found to form a partial subcomplex, we decided to investigate if it can bind to EcR in the absence of NELF-B (Fig. 1C). We depleted NELF-B subunit from the protein nuclear extract of *Drosophila* S2 cells and performed immunoprecipitation using antibodies against NELF-A, NELF-B and EcR. We observed that NELF-B depletion does not impair the interaction between NELF-A and EcR thus demonstrating an ability of a partial NELF-A-containing complex to interact with the ecdysone receptor. We observed only weak interaction between EcR and NELF-E inside NELF-A partial complex, which is in line with the fact that this interaction is weak even in normal conditions.

Thus, basing on the results of depletion experiments we can assume that the interactions of NELF-B and NELF-E with EcR, that we observed in co-immunoprecipitations provided in Fig. 1A, are in fact indirect and mediated by NELF-A, as a part of a complete or partial NELF complex.

### The rate of abortive transcription at the proximal regions of 20E-dependent genes decreases upon knockdown of NELF subunits

As NELF is a major factor driving the *Drosophila* Pol II transcriptional pause, we presumed it may also be responsible for Pol II pausing at the 20E-dependent genes^31^. Pol II pausing has previously been observed at most 20E-dependent promoters even in their uninduced state^1,2^. To evaluate the role of NELF in Pol II pausing at uninduced 20E-dependent genes, we decided to observe a functional marker of pausing—the rate of abortive transcription. We analyzed a previously published GRO-Seq dataset that measured 5′ proximal transcription in cells concomitantly treated with dsRNAs against NELF-B and NELF-E^29^. We specifically selected genes that can be induced upon 1-h treatment with 20E^1,2^. The mean GRO-Seq signal was plotted +/− 500 b. p. around transcription start sites (TSSs) of 20E-dependent genes both in mock and NELF-B/NELF-E dsRNA-treated cells (Fig. 1D). We observed a significant decrease in GRO-Seq signals proximal to TSSs of 20E-dependent genes in uninduced cells upon knockdown of NELF subunits demonstrating the role of NELF in Pol II pausing.

### RNA interference-mediated knockdown of NELF subunits decreases the rate of 20E-dependent transcription induction in *Drosophila* S2 cells

To evaluate the functional role of NELF in productive transcription of 20E-dependent genes, we performed knockdowns of each of four NELF subunits (Fig. 1E and Fig. S1). *Drosophila* S2 cells were treated with dsRNA for five days and then 20E-dependent transcription was induced by incubating the cells with 0.3 μM 20E for 1 h. We observed a significant decrease in inducible 20E-dependent transcription for 7 out of 8 tested genes. More importantly, knockdowns of each of four NELF subunits had a very similar effect on 20E-dependent transcription in comparison to the control (with a lower effect of NELF-E knockdown). We estimated the values of Pearson’s correlation for each pair of NELF subunits knockdowns and found them to be strongly positive (Fig. 1F). This demonstrates that the complete NELF complex is essential for the 20E-dependent genes to reach their induced transcription state.

The effect of individual NELF subunit knockdowns was also evaluated for uninduced *Drosophila* S2 cells, where 20E-dependent genes are inactive (Fig. S5). NELF subunits depletion caused mild but significant decrease in uninduced transcriptional level of *eip75a* and *eip78c* genes, which are characterized with a higher level of basal transcription in comparison to other tested 20E-dependent genes. We did not observe an increase in a basal transcription of the tested 20E-dependent genes upon knockdown of NELF subunits, comparable with their induced level of transcription. Thus, NELF factor does not work as an active transcriptional repressor of 20E-dependent genes in *Drosophila* S2 cells.

Altogether, our results reveal that NELF is important for the induced transcription of 20E-dependent genes and participates in the maintenance of their uninduced state by means of a Pol II pause.

### NELF is present at the promoters and enhancers of 20E-dependent genes both in their induced and uninduced states

Next, we investigated direct participation of NELF subunits in transcriptional response of 20E-dependent genes by exploring their binding to genes regulatory regions. We performed ChIP-Seq analysis using antibodies against NELF-A, NELF-B, or NELF-E subunits in 20E- or DMSO-treated cells (Fig. 2A). Antibodies against Rpb3 were also used to mark Pol II binding. The binding of NELF subunits were explored specifically on a set of rapidly induced 20E-dependent genes^1^.

We found that an enrichment of NELF subunits at the promoters of rapidly induced 20E-dependent genes is several times higher than NELF enrichment at the promoter of the averaged *Drosophila* gene (Fig. S5). We observed no change in binding of NELF subunits with 20E-dependent promoters before and after 20E treatment. These results corroborate our functional data, demonstrating the importance of NELF for induced response of 20E-dependent genes and maintenance of their uninduced, Pol II paused state. Simultaneous sorting of our ChIP-Seqs by NELF-A binding level demonstrated a positive correlation between amount of NELF subunits and Pol II bound at the 20E-dependent promoters (Fig. 2A). Thus, NELF may be recruited at Pol II-bound sites of 20E-dependent genes to restrain RNA polymerase II in a stable or temporal manner (i.e. during uninduced or induced transcriptional state).

Observations from NELF-averaged binding at 20E-dependent genes are reproduced at the level of an individual 20E-dependent gene (Fig. 2B). The distribution of NELF subunits at the *dhr3* and *hr4* loci, two of the most rapidly 20E-induced genes, is very well correlated with the profile of Rpb3. NELF subunits binding at the *dhr3* and *hr4* promoters increased upon 20E treatment, demonstrating involvement of NELF in an induced response of these genes. The level of NELF subunits at the *dhr3* promoter in its induced state was approximately two times higher than at the *hr4* promoter (the same as the level of Rpb3). These promoters represent an illustrative example of a strong correlation between chromatin-bound level of NELF factor and Pol II pause (a promoter-bound level of Pol II). The *hr4* promoter is in a less paused state and characterized with fewer amount of NELF and Pol II than *dhr3*. This may be the reason why we did not observe a change in an induced transcriptional response of the *hr4* gene upon the knockdown of NELF subunits (see the Fig. 1E). The presence of NELF subunits at the regulatory regions of *hr4* gene shows NELF involvement in induced transcription of this gene, but the lack of functional response on NELF knockdown demonstrates that Pol II pausing is not a critical regulatory step in this particular case^32^.

Close inspection of NELF distribution along individual 20E-dependent genes revealed the presence of NELF subunits (especially NELF-A) not only at promoters but also within gene bodies. This fact did not appear during investigation of averaged NELF profiles because they were calculated using coding regions of genes while some NELF subunits were found present at the sites located in the introns. To reveal whether these sites are intronic enhancers, we compared NELF subunits profiles with ChIP-Seqs of FLAG-EcR and CBP/Neijre, the ecdysone receptor and the major histone acetyltransferase marking enhancers, respectively. We indeed observed the presence of EcR and CBP/Neijre at NELF-bound sites at the bodies of the *dhr3* and *hr4* genes. To evaluate whether NELF is present in general at the enhancers of the 20E-dependent genes we plotted the average level of NELF subunits at the sites of enhancers (Fig. 2C). All three investigated subunits of NELF were found to bind enhancers of 20E-dependent genes, with NELF-A demonstrating slightly stronger binding. As the enhancers are bound by EcR, we presume these genomic sites to be the location of the observed protein interaction between NELF-A and EcR.

To verify the specificity of NELF enrichment at 20E-dependent promoters and enhancers, we plotted an averaged profile of NELF binding for the transcriptional end sites (TESs) of 20E-dependent genes (Fig. S7). We detected no enrichment of NELF subunits at the TES, thus proving the significance of their presence at the promoters and enhancers.

### NELF-A binding at 20E-dependent promoters corresponds to EcR-bound sites while NELF-B and NELF-E binding is shifted towards Pol II-bound sites

Upon close examination, we observed that at individual 20E-dependent promoters NELF-A binding does not exactly match binding of the NELF-B and NELF-E subunits (Fig. 2D). Instead, NELF-A binding is shifted towards EcR and CBP/Neijre-bound sites, which are located upstream the TSS (and presumably correspond to a closely located enhancer). Transcription induction provokes an increase in NELF-B and NELF-E binding at Pol II-bound sites of 20E-dependent genes. On the contrary, the level of NELF-A subunit remains similar after transcription induction and it does not shift towards the Pol II peak upon induction. This result demonstrates that the NELF-A subunit has a stronger association with EcR regulatory regions than the NELF-B and NELF-E subunits. This corroborates our current co-immunoprecipitation data, which showed interaction of a partial NELF-A-containing complex lacking NELF-B with EcR.

### Depletion of NELF subunits reduces promoter-bound level of RNA polymerase II both in the induced and uninduced states of 20E-dependent genes

To reveal the molecular role of NELF in transcription of 20E-dependent genes, we performed knockdowns of NELF subunits using RNA interference (Fig. 3A, B). It was challenging to obtain a sufficient reduction in the DNA binding level of individual NELF subunits upon their knockdown. This may result from an excess of NELF in the cell and its highly effective recruitment to genome targets. Therefore, we simultaneously treated cells with dsRNA against the NELF-A and NELF-B subunits. This resulted in considerably decreased intracellular protein levels of the NELF-A and NELF-B subunits and in reduction of transcription of corresponding genes (Fig. S1). We presumed that this double treatment impaired the state of both the partial and complete forms of NELF complex. Similarly, double knockdown of NELF-B and NELF-E subunits was previously employed to investigate the role of NELF in abortive transcription in *Drosophila* S2 cells^29^.

We observed a stronger decrease in NELF-B binding compared to NELF-A and therefore we used the reduction in the NELF-B level to select genes with impaired NELF binding. Thus, we chose 9 genes with NELF-B binding reduced more than 2 times upon NELF-A/NELF-B knockdown (Fig. 2A) to further investigate NELF contribution to 20E-dependent transcription.

We observed a decrease in Rpb3 binding at 20E-dependent promoters upon double NELF RNAi in the uninduced state of genes (Fig. 3C). This result indicates that NELF is responsible for Pol II pausing at 20E-dependent genes in their uninduced state. NELF reduction impairs the pause, which results in a loss of Pol II and a decrease in abortive transcription (Fig. 1D). Three genes with the highest 20E-dependent induction level (*dhr3, e23, cg3394*) demonstrated both the high level of NELF binding and the rate of its decrease upon NELF subunits knockdown (Table S2). It is interesting, that RNA inference of NELF subunits in uninduced states of these genes led not only to decrease in abortive transcription but also to significant increase in productive transcriptional level (Fig. S5). This effect means that at least on some 20E-dependent genes in their uninduced state NELF restrains the transcription elongation process (which is a feature of Pol II pausing).

The molecular role of NELF in induced transcription of 20E-dependent genes is less clear. We detected a decrease in promoter-bound Rpb3 upon NELF RNAi after 20E-dependent transcription induction. At the same time, we observed much less decrease in the level of phosphorylated Pol II isoforms (Fig. 3C). Thus, we concluded that the relative level of Pol II phosphorylation (both at the CTD Ser2 and Ser5) on 20E-induced promoters increases after NELF-A/NELF-B knockdown. We hypothesized that the NELF knockdown could lead to the problem with the formation of productive Pol II elongation complexes.

The most well-described driver of eukaryotic Pol II elongation through chromatin is the DSIF factor^33^. We decided to check whether binding of the Spt5 subunit of DSIF to Pol II is abrogated by NELF depletion (Fig. 3D). Indeed, we detected a significant decrease in promoter-bound Spt5 at 20E-dependent genes both in their induced and uninduced states upon NELF subunits knockdown. One possible explanation for the decrease in DSIF binding upon NELF depletion is a reduction in overall promoter-bound Pol II, as DSIF is recruited to chromatin through a direct interaction with Pol II^17,34^. However, we suppose that there is an additional NELF-dependent loss of DSIF at 20E-dependent genes. Close comparison of the decrease in median levels of Rpb3 (Fig. 3C) and Spt5 (Fig. 3D) at 20E-dependent promoters demonstrates that reduction of Spt5 binding exceeds changes in Rpb3 level, especially in the induced state. The median decrease in Rpb3 binding does not exceed two times, while Spt5 reduction is about three times. To demonstrate that NELF depletion does not influence the global level of Rpb3 and Spt5 binding we plotted an averaged profile of these proteins at 20E-dependent promoters, which demonstrates no reduction in NELF binding upon NELF depletion (Fig. S2). We also tested that the decrease in 20E-induced transcription upon NELF depletion is not a result of NELF-dependent loss of EcR (Fig. S8). We observed no decrease in EcR binding upon NELF-A/NELF-B double knockdown at the 20E-dependent promoters, which showed the strongest decrease in NELF binding.

The molecular effects observed in the averaged profiles of 20E-dependent genes impaired by NELF depletion are reproduced at individual promoters (Fig. 3E). We detected a considerable reduction in promoter-bound Pol II in the uninduced state of the *eip78c* and *cg1607* genes upon NELF depletion. The decrease in Rpb3 binding in the induced state of these genes was less prominent. On the contrary, we detected a considerable reduction in promoter-bound Spt5 upon NELF depletion in the induced state at *eip78c* and *cg1607*. The loss of Spt5 binding in the 20E-treated condition exceeded the reduction in Rpb3 binding level.

To demonstrate the effect of NELF-A/NELF-B depletion on transcription from 20E-dependent promoters, we depicted the molecular state of the promoters in *Drosophila* S2 cells before and after 20E-dependent induction (Fig. 3F). In the uninduced state, NELF promotes Pol II pausing and abortive transcription. After 20E-dependent induction, NELF maintains temporal pausing of Pol II, which is important for the stabilization of Pol II-DSIF complex. NELF depletion leads to loss of DSIF in the Pol II elongation complex, resulting in a lower rate of inducible transcription.

### NELF prepares 20E-dependent genes for upcoming transcription via Pol II pausing during the larva-to-pupa transition but not in embryogenesis

Ecdysone hormone is the main driver of *Drosophila* development. Its production is stimulated several times during development, which activates transcription of genes responsible for various vital decisions^35^. Among them are embryonic tissue specialization, larva molting, autophagy of some cell types and growth and differentiation of others during metamorphosis, pupal cuticle formation, etc^36^. A portion of genes that are directly induced with 20E are activated multiple times during development as they are transcription factors that are indispensable for the time-directed development of the organism^37^. We decided to dissect the mechanisms that regulate the transcription of 20E-dependent genes at different stages of development and determine the role of NELF factor in it.

We concentrated on two important developmental transitions: early hours of embryogenesis when the ecdysone cascade induction coincides with cell-type specialization and the larva-prepupal transition when an increase in 20E initiates formation of prepupa (Fig. 4A). Thus, we collected embryos at different stages (2–4 h, 20E-dependent genes are inactive; 6–8 h, transcription is initiated, and 10–12 h, they are actively transcribed). We also collected larvae approximately 12 h before pupariation, while they were actively feeding (corresponding to PS1-3), and 0–1 h after puparium formation (APF) (the former has a low level of 20E and the latter has active transcription of ecdysone-cascade genes). To validate the stage selection for our collected material we measured the transcriptional rate of *dhr3* and *hr4* 20E-dependent genes and demonstrated their active transcription during each developmental stage with a high level of 20E hormone production^1,27^ (Fig. S3).

We performed ChIP-Seq with material from the selected developmental stages using antibodies against Rpb3, Pol II phosphorylated at Ser2 in the CTD, and the NELF-A subunit (Fig. 4B). We chose 12 genes out of a set of rapidly induced 20E-dependent genes that are active during 10–12 h after egg laying (AEL) and 0–1 h APF, for further analysis (Fig. 4B)^1,27^.

Averaged profiles of Rpb3 binding at the promoters of stably induced 20E-dependent genes demonstrate the absence of Pol II binding during early hours of embryogenesis (Fig. 4B). Transcription induction of 20E-dependent genes during 10–12 h AEL coincides with an increase in both phosphorylated and promoter-bound Pol II. During later developmental stages when 20E-dependent genes are inactive (larva PS1-3) we still detected a high level of total Pol II with a decrease in Pol II phosphorylation, indicating that in larva 20E-dependent promoters are in Pol II paused state. Active transcription of 20E-dependent genes during the larva-to-prepupa transition is accompanied by an increase in Pol II phosphorylation but not its total promoter-bound level. Averaged profiles of NELF-A ChIP-Seq correlate very well with the amount of Rpb3 at 20E-dependent promoters. NELF-A does not bind 20E promoters until 10–12 h of embryogenesis but remains bound during their transcriptionally inactive state in larva PS1-3. Based on our results we presume that NELF participates in induced response of 20E-dependent genes both in embryogenesis and in prepupa as well as in organization of the Pol II paused state at the promoters in larva during temporal inactivation of 20E genes.

We used GAP genes (*btd, cad, kn, gt, hb, hkb, kr, slp1, tll*) as a set of control genes that have alternative developmental transcriptional profiles, as their transcription is induced during the first hours of embryogenesis and responsible for embryo segmentation (Fig. S9). Averaged profiles of our ChIP-Seqs on promoters of these genes demonstrate that Pol II and NELF bind only during embryogenesis. We did not detect the presence of Pol II and NELF at these genes in larva PS1-3, when these factors were found to bind 20E-dependent promoters. It is interesting to note that NELF-A does not bind GAP genes in early embryos where these genes are actively transcribed and loaded with Ser2-phosphorylated Pol II. NELF-A is later recruited at the promoters of these genes (6–8 h AEL) and its recruitment coincides with a decrease in the phosphorylation of Pol II (and an increase of its total amount on the promoter possibly due to Pol II pausing). Thereby, NELF is not involved in induced response of GAP genes but seems to drive a certain stage of their inactivation through decreasing phosphorylation of Pol II at their promoters and stalling the enzyme.

The change in NELF-A and Pol II binding on promoters of 20E-dependent and GAP genes observed in the averaged ChIP-Seq profiles is well reproduced at the individual genes (Fig. 4C). The most notable difference is the presence of a considerable amount of NELF-A and Pol II at the promoters of 20E-dependent but not at the GAP genes during larva PS1-3 stage. This can be attributed to Pol II pausing as a temporal mechanism of inactivation of the genes whose transcription is induced multiple times during development.

To find out whether the complete NELF complex is involved in induced response of 20E-dependent genes both in embryogenesis and during larva-to-pupa transition, we performed ChIP analysis using antibodies against NELF-B and NELF-E, in addition to NELF-A (Fig. 4D). A significant level of the enrichment was demonstrated for all tested NELF subunits at the promoters of 20E-dependent genes (with a strong positive correlation in a presence of NELF-A, NELF-B and NELF-E during 10–12 h of embryogenesis, larva PS1-3 and 0–1 h APF) (Fig. S10). As the presence of all of the NELF subunits were previously shown to be important for establishing of Pol II paused state, we draw a conclusion that the complete NELF complex is involved in Pol II pausing at 20E-dependent promoters in *Drosophila* development^17^.

We presume that transcriptional activation of 20E-dependent genes proceeds through different mechanisms during embryogenesis or metamorphosis, as visualized in our model (Fig. 4E). In the early hours of embryogenesis, NELF and Pol II do not bind 20E-dependent promoters. Transcriptional induction of 20E-dependent genes occurs through a mechanism of Pol II recruitment. However, after transcriptional inactivation of 20E-dependent genes in late embryogenesis, Pol II and NELF are not removed from the promoters of these genes and their transcription is inactivated via Pol II pausing by NELF. Thereby, transcriptional induction of 20E-dependent genes during metamorphosis does not include Pol II recruitment but represents the stimulation of a pause release through Pol II phosphorylation.

## Discussion

In our recent work we revealed an interaction between the EcR/Usp and NELF-A subunit of the Negative elongation complex^3^. Our present study was aimed to describe the relationships which exist between NELF and EcR and its role in 20E-dependent transcription.

### A partial NELF complex is present at the EcR bound sites

We first demonstrated that EcR indeed interacts with the complete NELF complex, with a stronger interaction between EcR and the NELF-A subunit. In the depletion experiments we observed the ability of EcR to bind also a partial NELF-A containing complex, lacking NELF-B. This result provides a new perspective on the mechanism of NELF loading at promoter-bound Pol II.

We assume the possibility of a step-by-step assembly of the NELF complex at 20E-dependent loci. An observed interaction between NELF-A-containing partial complex and EcR may occur at the EcR-bound enhancers and be an initial step of NELF recruitment. The formation of a NELF complex is completed at the Pol II bound sites by the recruitment of other subunits. Indeed, a high level of the NELF-A subunit on the regulatory regions of 20E dependent genes coincides with EcR binding, while the binding of other NELF subunits presents distinct features. NELF-B and NELF-E are almost scarce at EcR bound sites, but precisely colocalize with Pol II. The very good question is how partial NELF is redistributed from EcR-bound enhancers to the Pol II-bound sites. Most likely, it is a result of three-dimensional interaction between 20E-regulatory regions. But to test this hypothesis, additional experiments are obviously needed.

Interestingly, the mechanism of NELF functioning has been the subject of many detailed studies, while the process of its recruitment to a regulated gene is not well understood. We searched the literature to understand whether the recruitment of the NELF complex to its targets through interaction with nuclear receptors is widespread or not. It turned out that the interaction of EcR and NELF is not the only example. It was previously demonstrated that the NELF-B subunit of the human NELF complex interacts with the estrogen receptor (ER) and represses ERα-mediated transcription^38^. Another component of the murine NELF complex, the NELF-C/D subunit, directly interacts with androgen receptor (AR) as well as represses an androgen signal‐transduction cascade by decreasing the stability of AR^39^.

### NELF organizes the Pol II pause at the uninduced 20E-dependent genes

NELF plays a dual role in the transcriptional regulation of 20E-dependent genes described in the present study. On one hand NELF participates in Pol II pausing at uninduced state of these genes, and on the other hand it is involved in their induced transcriptional response. We have demonstrated that NELF depletion causes the disruption of Pol II pause during the uninduced state of 20E genes, which is confirmed by a decrease in Pol II binding to the promoters and in the level of abortive promoters-proximal transcription. Some of 20E-inducible genes show a significant increase in their productive transcription in the uninduced state upon NELF subunits knockdown. This effect very well corresponds to the behavior of *hsp70* gene. This classical *Drosophila* Pol II pausing model gene demonstrates three times increase in its productive transcription elongation upon NELF knockdown, which is assumed to be an evidence of NELF role in restraining of the Pol II elongation process^9,40^. However, NELF knockdown doesn’t result in the activation of the 20E genes to their fully induced transcriptional levels. NELF does not function as an active repressor of 20E-dependent genes. We suggest, that its presence at these genes in their uninduced state prepares them for the forthcoming transcription via Pol II pausing, which coordinates their response to 20E.

The recently described structure of the Pol II-DSIF-NELF complex has shed light on the molecular function of NELF in Pol II pausing^17^. NELF binds the polymerase funnel, bridges two mobile polymerase modules, and contacts the trigger loop, thereby restraining Pol II mobility that is required for pause release. All of these NELF properties are in line with its role as a pausing factor, which prepares genes for the forthcoming transcription. Much less clear is how NELF restraining of Pol II is integrated in induced transcription of the genes^14,41^.

### NELF promotes formation of the Pol II elongating complex by stabilizing the Pol II-DSIF interaction

Our present work demonstrates the inability of 20E-dependent genes to reach their full inducible level upon NELF depletion. We link the given function of NELF with its role in stabilization of a complex between DSIF and Pol II in the early moments of elongation of 20E-dependent genes. It is known that in DSIF-depleted cells, a fraction of Pol II molecules is dislodged during elongation, thus decreasing the number of Pol II complexes that complete the transcription cycle^33^. Another study demonstrated that DSIF is globally required for Pol II accumulation at the promoters and for the majority of productive mRNA synthesis^42^. Thus, we think that the presence of NELF on the promoters of actively transcribed genes allows it to indirectly influence the elongation of Pol II, affecting the stability of the complex between DSIF and Pol II inside temporarily paused Pol II complex. Although previously observed, the effect of NELF on the stabilization of the complex between Pol II and DSIF is not well-described. Early in vitro assays investigating DSIF and NELF loading revealed that the presence of DSIF in the Pol II complex is essential for NELF binding^34^. But the reverse impact was also observed: in low concentrations DSIF was unable to bind Pol II in the absence of NELF. In a more recent structural study, it was shown that a NELF-A subunit ‘tentacle’ contacts the DSIF factor within the Pol II-DSIF-NELF complex^17^. This direct interaction actually may stabilize the complex between DSIF and Pol II.

At the same time, the leading effect of NELF on the induced transcriptional response may be the stabilization of the temporary Pol II pause, which provides the necessary time for the formation of a productive elongation complex.

### NELF is involved in Pol II pausing of 20E-dependent genes in *Drosophila* development

20E-dependent genes are rather unusual in that they are activated repeatedly in development. A recent study by Uyehara and McKay compared the changes in EcR binding across the *Drosophila* genome during the larva-to-pupa transition in the wing imaginal discs^43^. They revealed a highly dynamic EcR binding to its target sites during development. However, comparing EcR binding in wing tissues and S2 cells of embryonic origin, they observed some common EcR binding sites. The presence of a constant set of genes that are induced by 20E in various tissues types was also reported by Shlyueva and colleagues^44^ in a study on ecdysone-dependent enhancers. But still, the processes that occur on promoters of these constant 20E targets at different stages of development were not characterized until now.

The present study deals specifically with a set of 20E constant targets, most of which are induced by 20E hormone in many cell types^45^. ChIP-Seqs of Pol II total, Pol II Ser2P and NELF during development allowed us to hypothesize a molecular mechanism driving transcription of 20E-dependent genes. According to our data, these genes are activated in embryogenesis by recruiting Pol II, retain bound Pol II on their promoters during larval stages, and are again activated in pupariation by stimulating release of the Pol II pause. We assume that NELF helps to maintain a Pol II pause at the promoters of 20E-dependent genes during *Drosophila* development, since NELF is responsible for the Pol II pause at 20E-dependent promoters in the cell culture. The presence of three out of four NELF subunits on 20E-dependent promoters at the larval stage, when these genes are inactive, but are preparing for the forthcoming induction, further confirms the involvement of NELF in the process of Pol II pausing.

The transient transcriptional pausing of 20E-dependent genes during larval development can be viewed as a type of transcriptional memory that allows genes to be easily reactivated^46^. It was shown, that in *Drosophila* embryo development the transcriptional memory plays an important role: transcription of gene in a daughter cell is activated faster and stronger if this gene was active in a mother nucleus^46^. The Pol II pausing was found to be a part of a mechanism implementing transcriptional memory—the transition of genes in the Pol II paused state during embryo development increases the likelihood of their activation^47^. Why a cell chooses transcriptional pausing for temporal inactivation of 20E-dependent genes remains to be seen—perhaps it preserves the competence of the tissue in which the ecdysone cascade has already been activated to repeat the activation in the course of metamorphosis.

Despite the multitude of previous studies on Pol II pausing mechanisms in early *Drosophila* development, the role of the NELF complex in these processes has mostly been overlooked^20,48^. Currently it seems that the role of this factor may go beyond spatiotemporal control of transcription activation—likely, NELF also impacts the quality of the produced transcripts, as well as contributes to transcription repression. We hope that the power of the available experimental techniques will make it possible to further dissect the role of NELF in these processes taking place at *Drosophila* developmental genes.

## Supplementary Information

Supplementary Figure.
