# Supplementary material for: The negative elongation factor NELF promotes induced transcriptional response of Drosophila ecdysone-dependent genes
Source: Sci Rep. 2021 Jan 8;11:172. doi: 10.1038/s41598-020-80650-1 (PMC7794308; doi:10.1038/s41598-020-80650-1)
Supplement: Supplementary file 1 — Supplementary Figure. [file 41598_2020_80650_MOESM1_ESM.pdf]

The negative elongation factor NELF promotes induced transcriptional response of *Drosophila* ecdysone-dependent genes

Marina Yu. Mazina[1], Elena V. Kovalenko[1], Nadezhda E. Vorobyeva[1]*

[1]Institute of Gene Biology, Russian Academy of Sciences, Moscow 119334, Russia

* Corresponding author (email to vorobyeva@genebiology.ru)

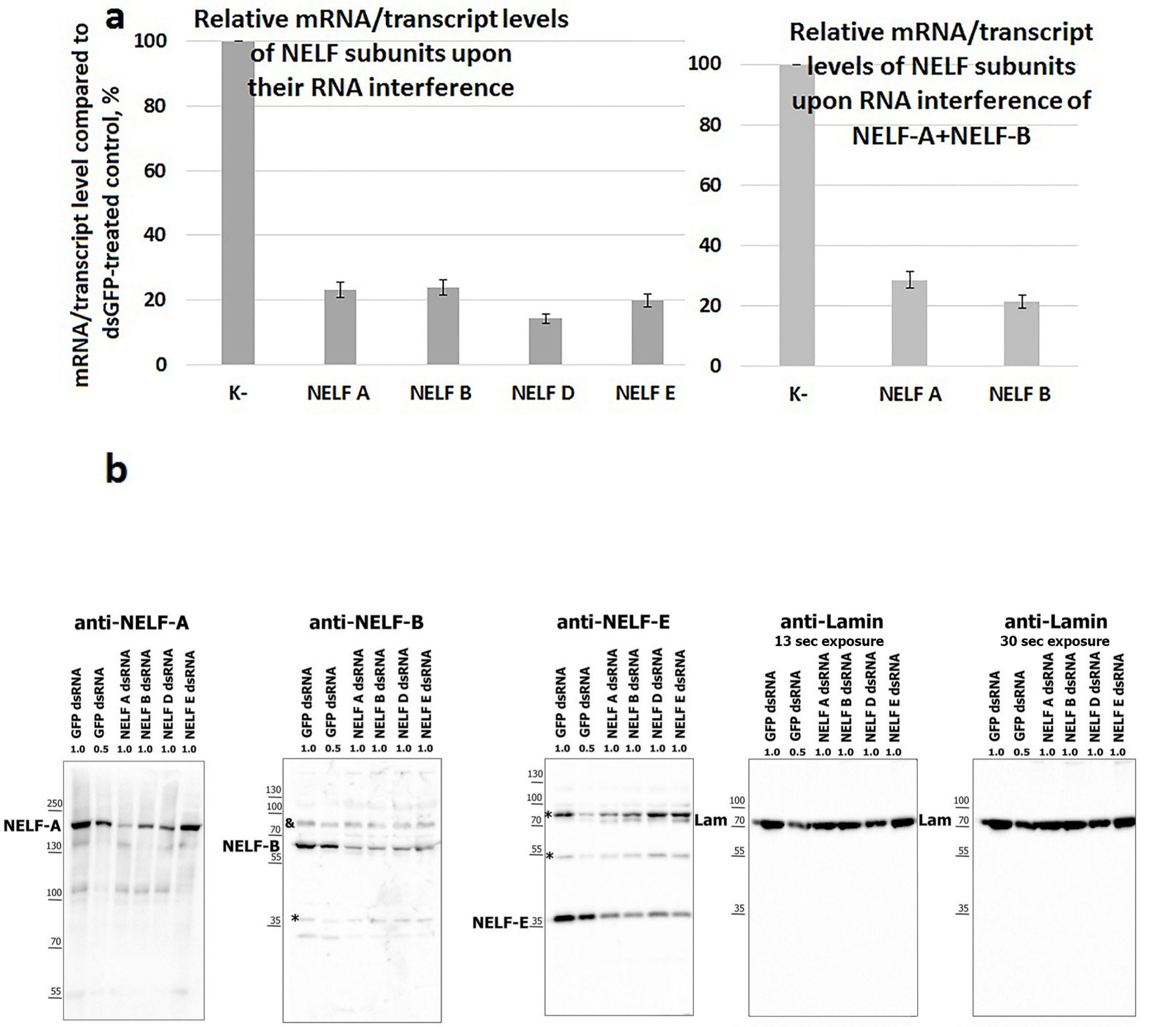

* Lines corresponding to unspecific antibody-derived background; & Line left from a previous stainig of this blot with anti-Lam

****The high specificity of antibodies against the Lamin did not allow us to register the background signal**

Supplementary figure 1. (a) The reduction in mRNA level of NELF complex subunits upon their RNA interference-mediated knockdowns. S2 *Drosophila* cells, treated with dsRNA corresponding to GFP transcript, were used as a control (K-). Transcriptional levels were assessed by qRT-PCR. The Y-axis units represent expression level of the subunit in the corresponding knockdown compared to subunit expression in K- in percents. Data are mean values from three independent experiments, error bars represent standard deviations. (b) The test of RNA interference-mediated knockdowns efficiency of NELF subunits. Protein extracts from S2 *Drosophila* cells, treated with dsRNA against NELF-A, NELF-B and NELF-E, and dsRNA to GFP transcript were loaded. The antibodies, used for blot staining, are marked on the left of the figure. Numbers above the figure represent the relative portion of a loaded fraction. Anti-tubulin staining was used as loading control.

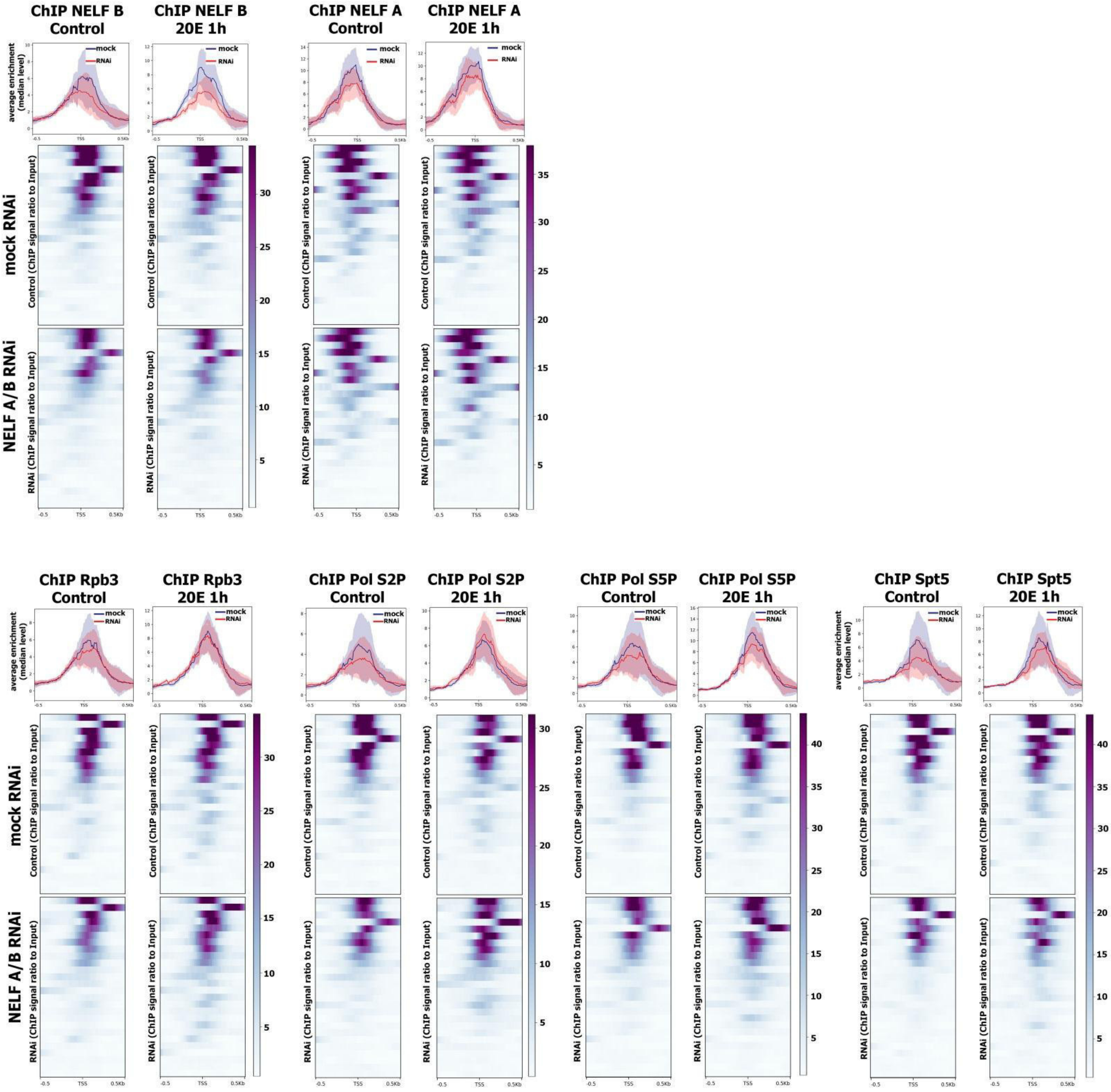

Supplementary figure 2. Average distribution of binding of NELF-B, NELF-A, Rbp3, Pol II, phosphorylated on Ser2 of CTD (Pol S2P), Pol II, phosphorylated on Ser5 of CTD (Pol S5P) and Spt5 on the transcription start sites (TSS) of 20E-dependent genes in *Drosophila* S2 cells upon 1h 20E induction (20E 1h) or in DMSO-treated cells (control). The cells were concomitantly treated with dsRNAs against NELF-B and NELF-A (NELFA/B RNAi) or dsRNA corresponding to GFP transcript (mock RNAi). Pile-up profiles were generated for the TSSs of 27 20E-dependent genes whose promoter regions demonstrated less than 2-fold decrease in NELF-B binding level upon treatment with dsRNA against NELF-A/NELF-B. Protein binding levels were estimated by ChIP-Seq as an enrichment (averaged profiles were generated as a median level). The standard error is displayed on the graphs as lighter area around the main line of the profiles.

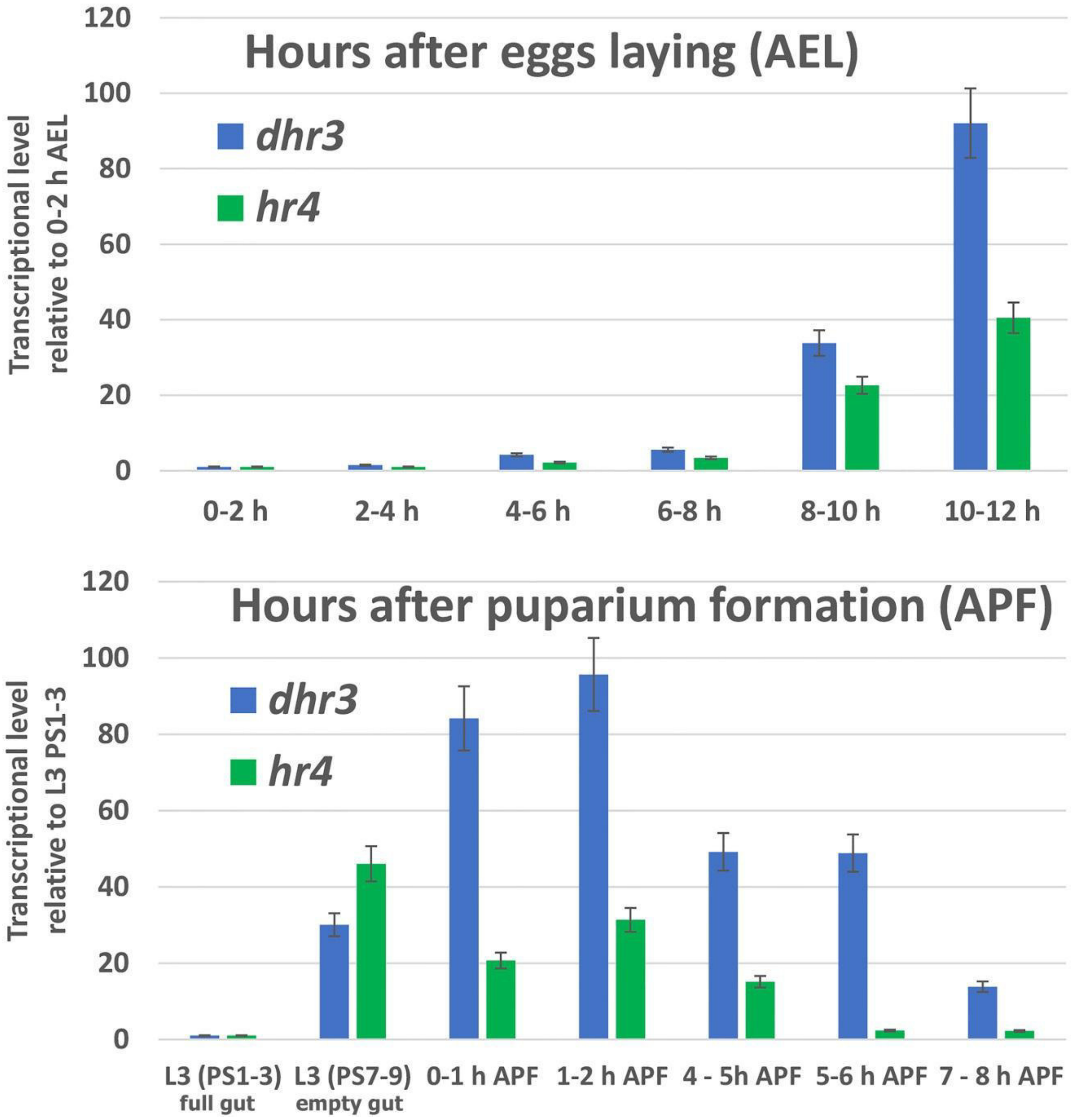

Supplementary figure 3. The transcription levels of *dhr3* (blue columns) and *hr4* (green columns) 20E-dependent genes during ontogenesis of *Drosophila* relative to their transcription during 0-2 hours after egg laying (AEL), for embryonic development, or relative to their transcription during L3 stage PS1-3 (full gut), for the stages after pupariation.

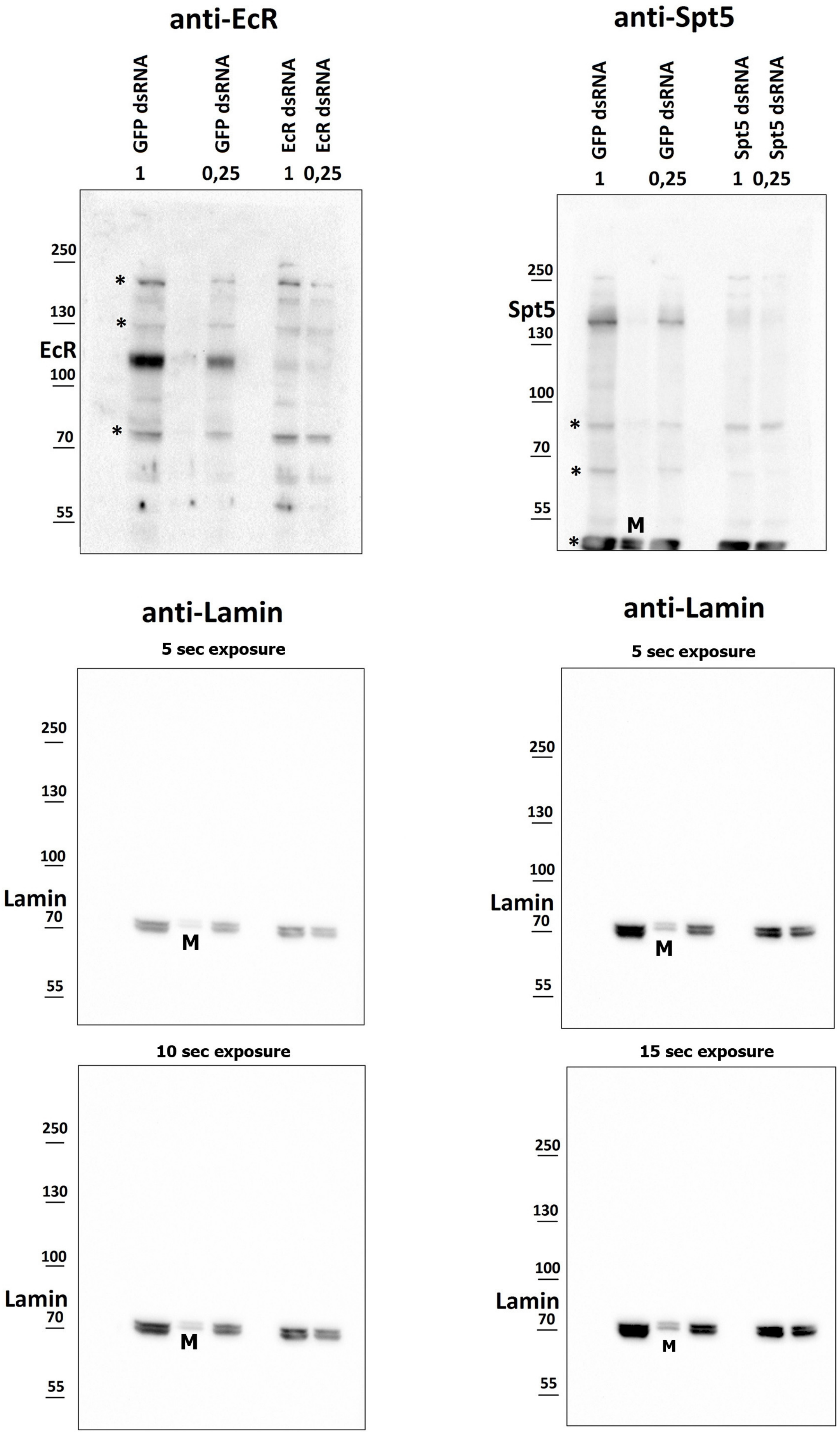

***Lines corresponding to unspecific antibody-derived background;**
M-control protein extract introduced into the well by mistake
****The high specificity of antibodies against the Lamin did not allow us to register the background signal**

Supplementary figure 4. The test of antibodies against EcR and Spt5. The depletion of corresponding proteins with RNA-interference was used to estimate the specificity of antibodies. Nuclear extracts from S2 *Drosophila* cells, treated with corresponding dsRNA, and dsRNA to GFP transcript were loaded. The antibodies, used for blot staining, are marked on the left of the figure. Numbers above the figure represent the relative portion of a loaded fraction. Anti-lamin staining was used as loading control.

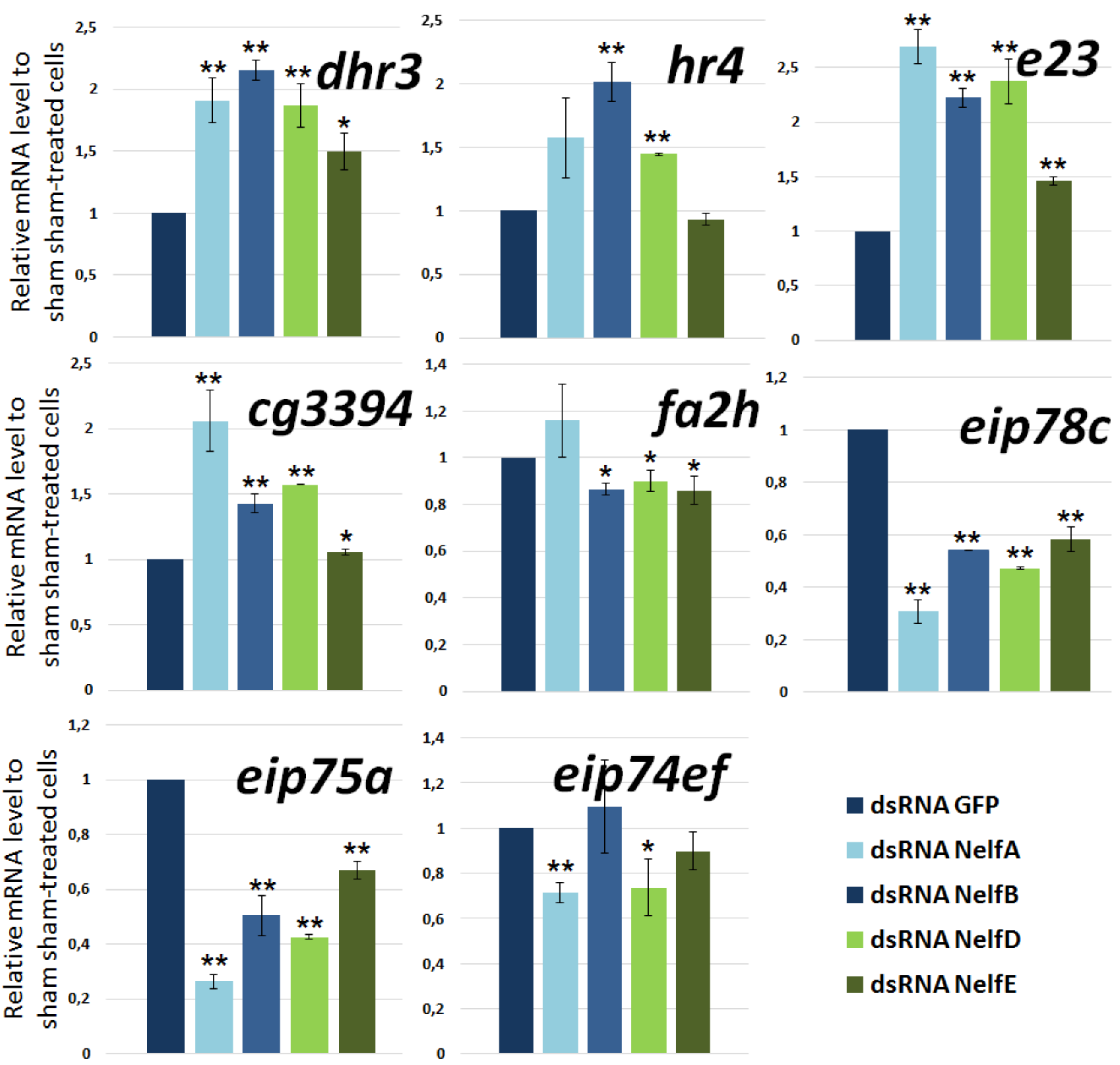

Supplementary figure 5. Relative mRNA levels of uninduced 20E-dependent genes in *Drosophila* S2 cells, treated with dsRNA corresponding to NELF-A, NELF-B, NELF-D and NELF-E subunits of NELF complex (dsRNA corresponding to GFP transcript in mock RNAi). Transcriptional levels were assessed by qRT-PCR. The Y-axis units represent transcriptional level of corresponding gene relative to mock RNAi treated cells, normalized on tubulin mRNA. Data are mean values from three independent experiments, error bars represent standard deviations. Asterisks indicate significance levels (Student's t-test), ** - p-value < 0.01, * - p-value < 0.05. Statistical analysis was performed relative to the transcription levels of the genes in GFP dsRNA treated S2 cells.

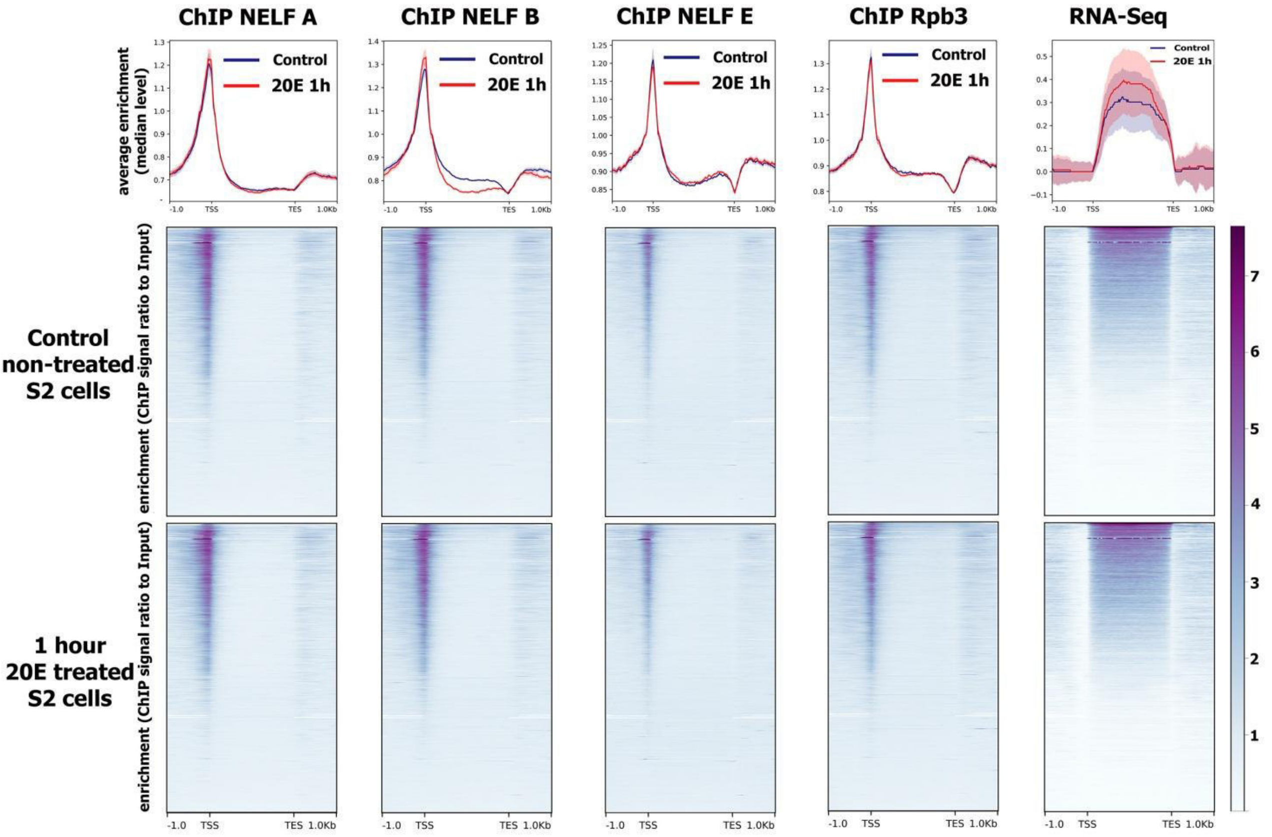

Supplementary figure 6. Average distributions of NELF-A, NELF-B, NELF-E and Rpb3 on all genes in *Drosophila* S2 cells after 1-hour treatment with 20E (20E 1h) or in non-treated cells (control). NELF-A, NELF-B, NELF-E and Rpb3 binding levels were estimated as an enrichment of NELF-A, NELF-B, NELF-E and Rpb3 ChIP-Seq signal over input DNA. Pile-up profiles were calculated as a median of NELF-A, NELF-B, NELF-E and Rpb3 binding levels. Average profiles were generated using metagene mode (introns were ignored). The standard error is displayed on the graphs as lighter area around the main line of the profiles.

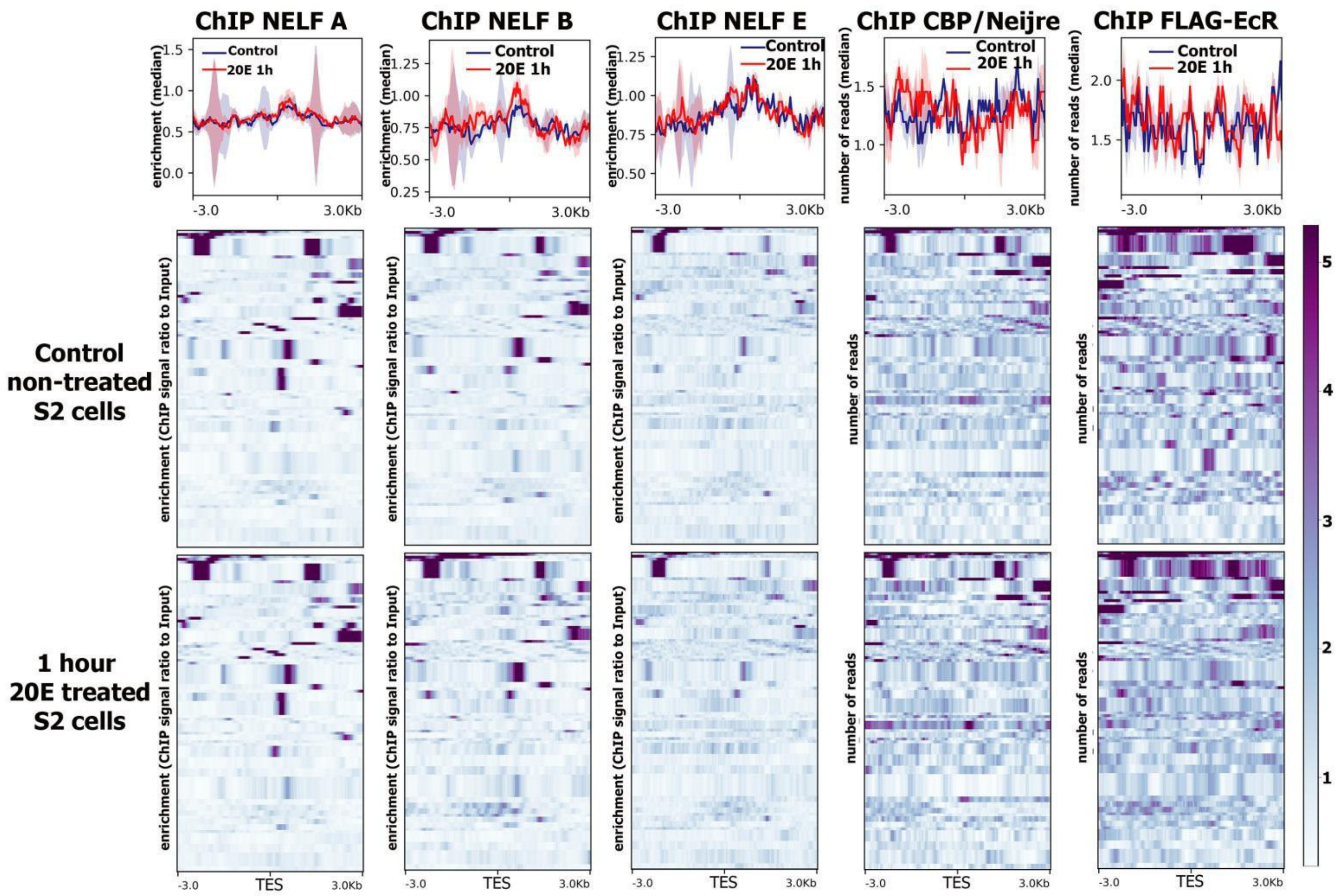

Supplementary figure 7. Average distributions of NELF-A, NELF-B, NELF-E, CBP/Neijre, FLAG-EcR binding and RNA-seq signal on transcription end sites (TESs) of 20E-dependent genes in *Drosophila* S2 cells after 1 hour treatment with 20E (20E 1h) or in non-treated cells (control). The ChIP-Seq data on FLAG-EcR binding were obtained in our previous work[1]. NELF-A, NELF-B, NELF-E, CBP/Neijre and FLAG-EcR binding levels were estimated as an enrichment of NELF-A, NELF-B, NELF-E, CBP/Neijre and FLAG-EcR ChIP-Seq signal over input DNA. Pile-up profiles were calculated as a median of NELF-A, NELF-B, NELF-E, CBP/Neijre and FLAG-EcR binding levels. The standard error is displayed on the graphs as lighter area around the main line of the profiles.

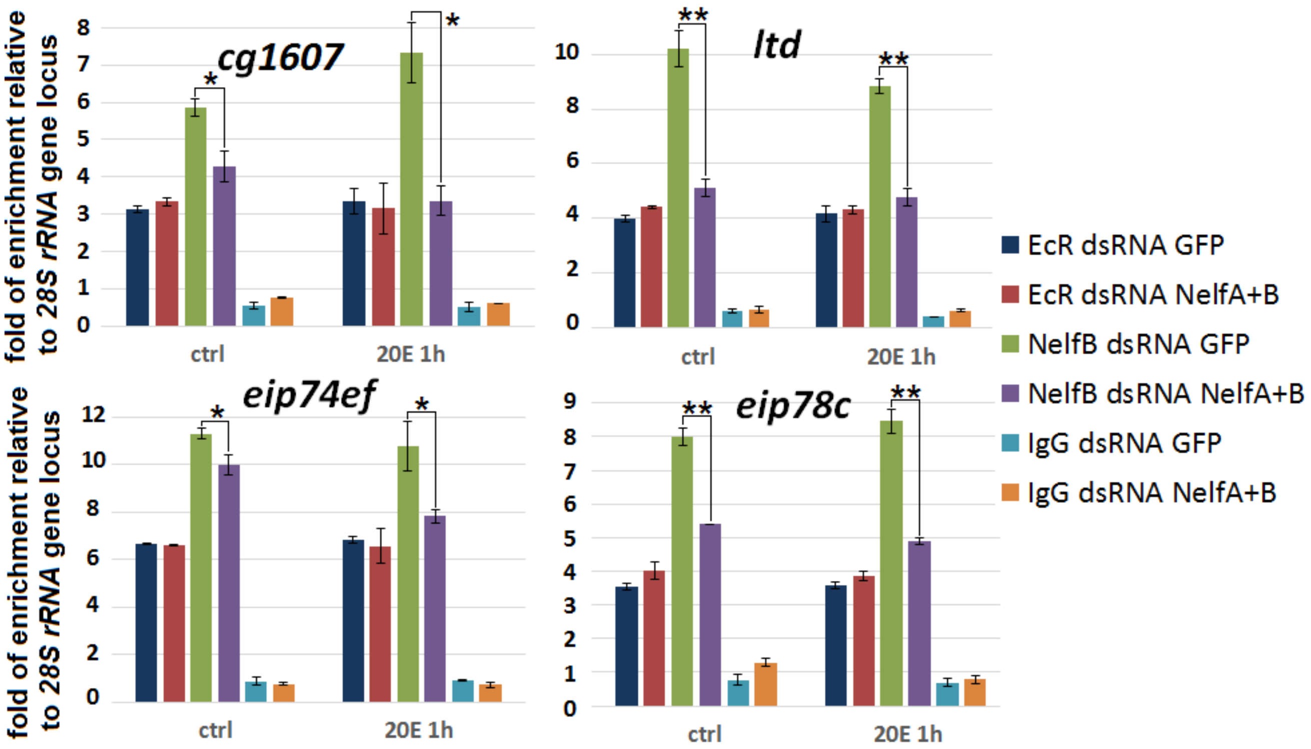

Supplementary figure 8. ChIP analysis of EcR and NELF-B binding to 20E-dependent promoters, which demonstrate the strongest reduction in NELF-B binding level upon NELFA+NELF-B knockdown, in 1-hour 20E-induced *Drosophila* S2 cells (20E 1h) relative to DMSO-treated (ctrl). The impact of NELF-A and NELF-B on EcR binding was estimated using RNA interference-mediated knockdown with corresponding dsRNA (GFP dsRNA was taken as a mock RNAi). The Y-axis units represent the fold of enrichment relative to the control region (28S ribosomal RNA gene locus). ChIP with a serum of non-immunized rabbits (IgG) was used to assess the specificity of antibodies. The data are mean values from three independent experiments, error bars represent standard deviations. Asterisks indicate significance levels (Student's t-test), ** - p-value < 0.01, * - p-value < 0.05.

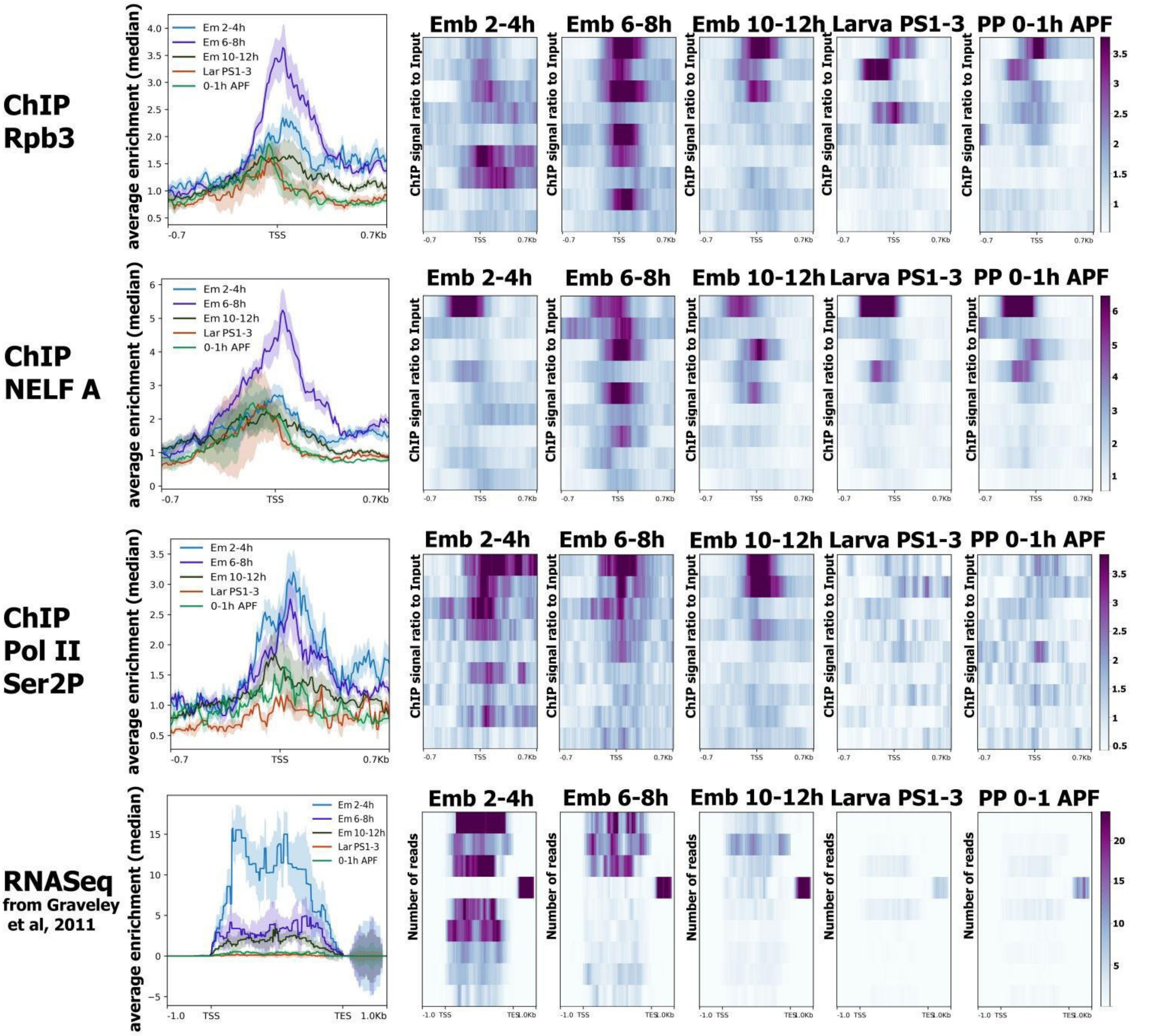

Supplementary figure 9. Average distribution of Rbp3, NELF-A and Pol II Ser2P binding on transcription start sites (TSS) of nine GAP genes at different developmental stages (*btd, cad, kn, gt, hb, hkb, kr, slp1, tll*). Protein binding levels were estimated by ChIP-Seq as an enrichment of ChIP signal to input. Averaged RNA-seq profiles were calculated for GAP genes on different developmental stages, using the data from [2].Pile-up profiles were calculated as a median of Rbp3, NELF-A and Pol II Ser2P binding levels. The standard error is displayed on the graphs as lighter area around the main line of the profiles.

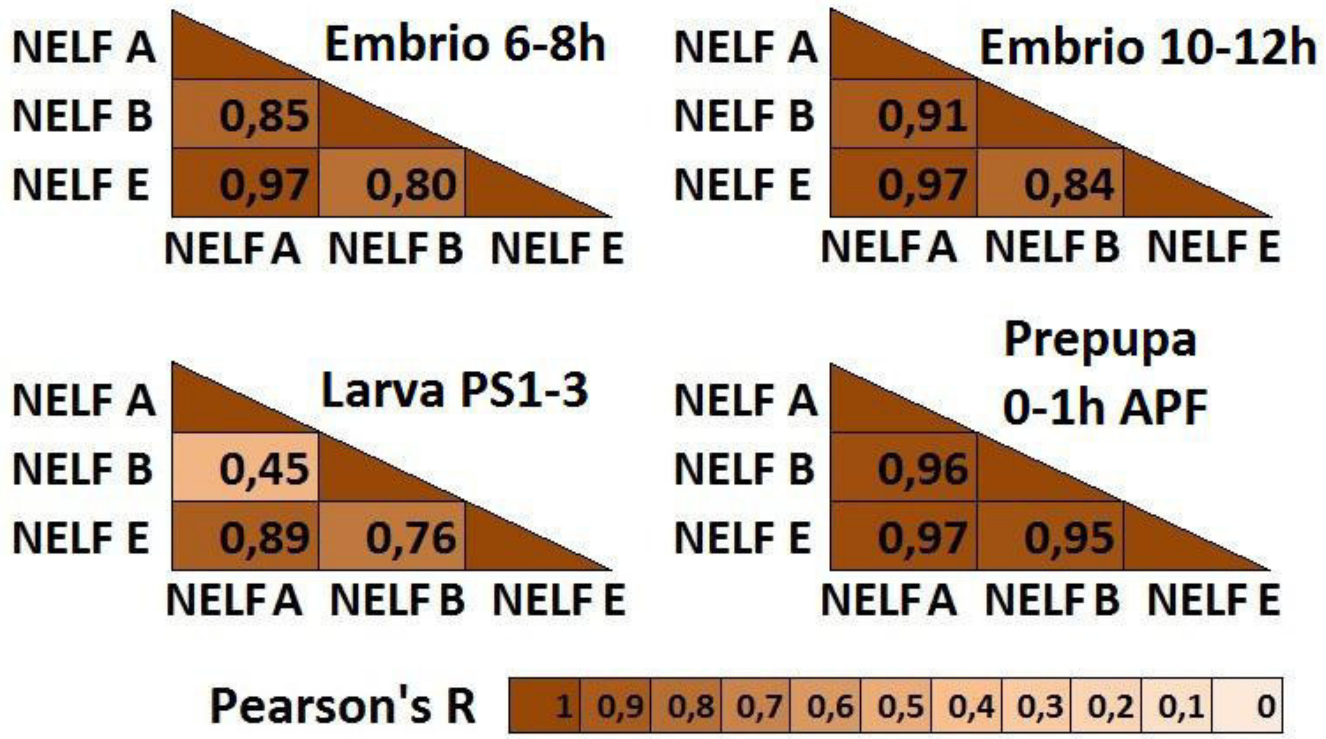

Supplementary figure 10. Pearson's correlation matrix for the NELF-A, NELF-B and NELF-E subunits binding to promoters of some stably induced 20E-dependent genes at different developmental stages of *Drosophila.* The list of the genes is the same as on Fig. 4D. The heat map reflects the indicated R values.

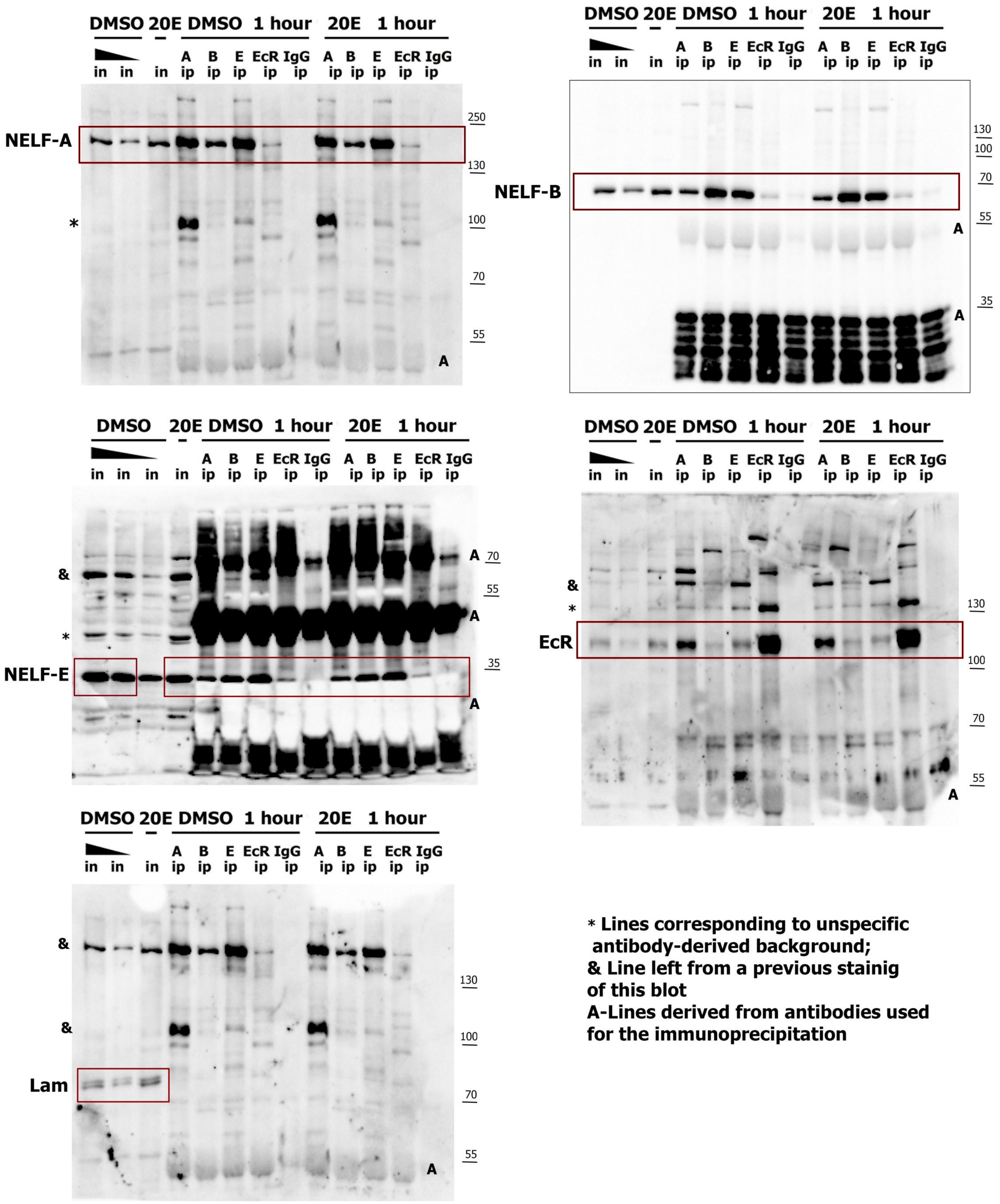

Supplementary figure 11. The original Western blots (full-size) from the Fig. 1A

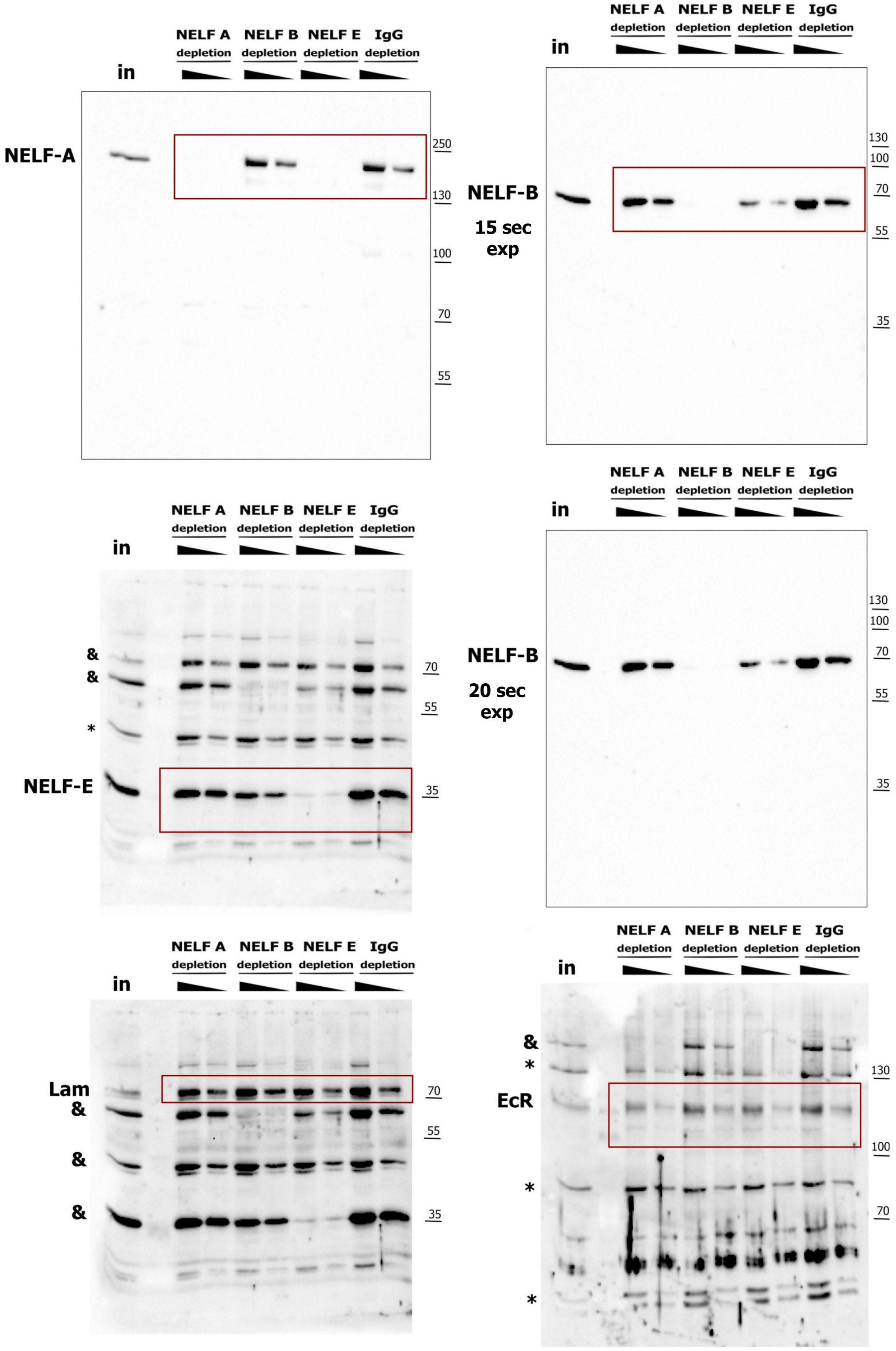

* Lines corresponding to unspecific antibody-derived background;

& Line left from a previous stainig of this blot

Supplementary figure 12. The original Western blots (full-size) from the Fig. 1B

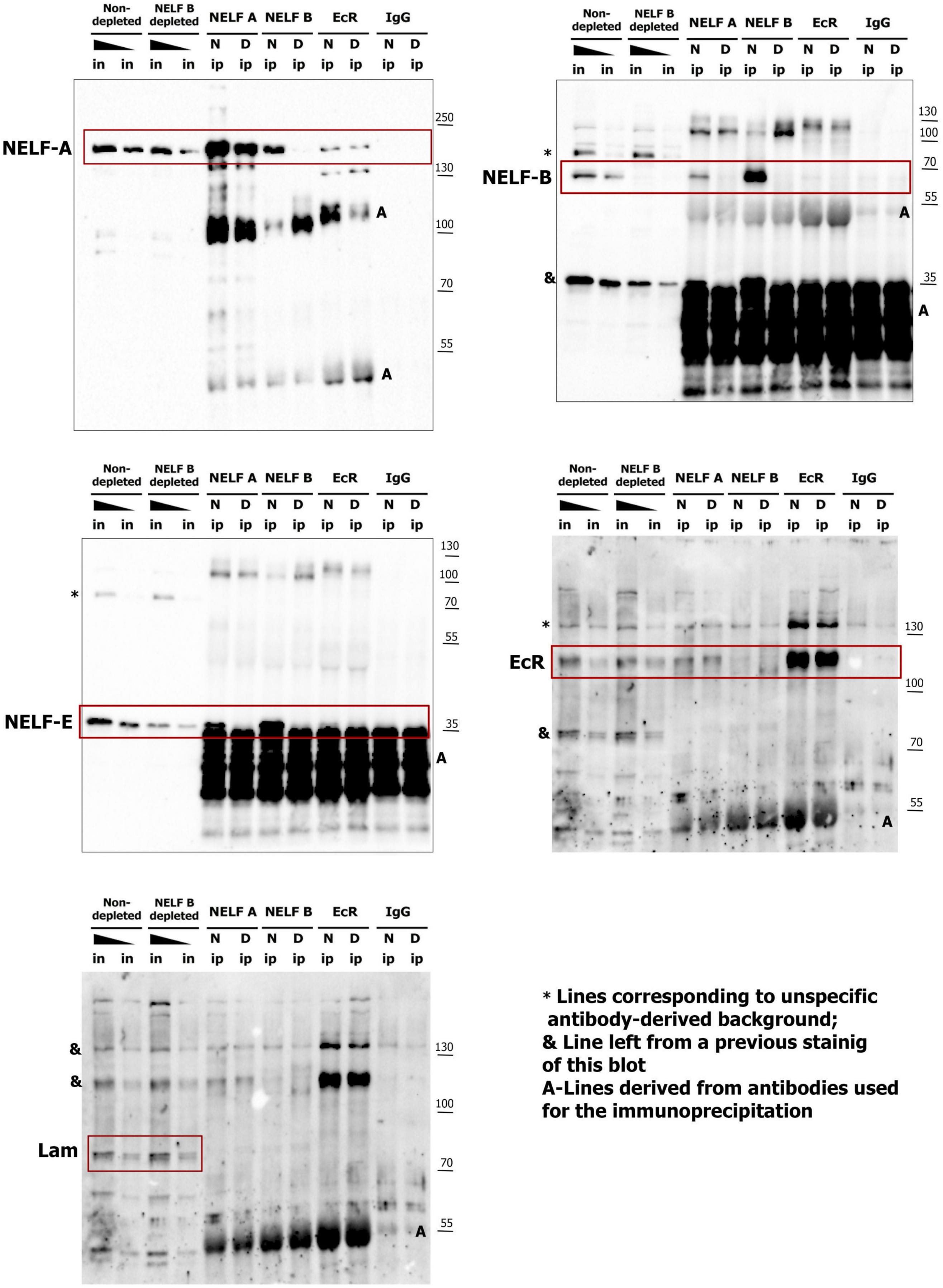

* Lines corresponding to unspecific antibody-derived background;
& Line left from a previous stainig of this blot
A-Lines derived from antibodies used for the immunoprecipitation

Supplementary figure 13. The original Western blots (full-size) from the Fig. 1C

Table. S1. List of genes induced by 20E 1hour treatment in S2 cells more than 2 times

Chrom	Start	End	Name	Score	Strand	ThickStart	ThickEnd	ItemRGB	BlockCount	BlockSizes	BlockStarts
**chrX**	1941939	2005462	NM_001038734.2	0	+	1997586	2002540	0	11	65,467,186,94,49,58,2824,149,1389,119,3003,	0,487,11341,25956,34632,44458,55642,58614,58831,60295,60520,
**chrX**	1941939	2005462	NM_001272227.1	0	+	1942864	2002540	0	12	65,467,186,94,49,58,980,2824,149,1389,122,3003,	0,487,11341,25956,34632,44458,51270,55642,58614,58831,60292,60520,
**chrX**	1992299	2005462	NM_001272228.1	0	+	1994624	2002540	0	7	436,497,2824,149,1389,119,3003,	0,2102,5282,8254,8471,9935,10160,
**chrX**	1992299	2005462	NM_001272229.1	0	+	1993222	2002540	0	6	1890,2824,149,1389,119,3003,	0,5282,8254,8471,9935,10160,
**chrX**	1941939	2005462	NM_001272230.1	0	+	1989922	2002540	0	12	65,467,186,94,49,58,190,2824,149,1389,122,3003,	0,487,11341,25956,34632,44458,47818,55642,58614,58831,60292,60520,
**chrX**	1941939	2005462	NM_001272231.1	0	+	1997586	2003572	0	11	65,467,186,94,49,58,2824,149,1389,122,3003,	0,487,11341,25956,34632,44458,55642,58614,58831,60292,60520,
**chrX**	1941939	2005462	NM_001272232.1	0	+	1997586	2004420	0	12	65,467,186,94,49,58,2824,149,1389,122,1093,1842,	0,487,11341,25956,34632,44458,55642,58614,58831,60292,60520,61681,
**chrX**	1941939	2005462	NM_001272233.2	0	+	1993222	2002540	0	11	954,186,94,49,58,1175,2824,149,1389,122,3003,	0,11341,25956,34632,44458,51075,55642,58614,58831,60292,60520,
**chrX**	1941939	2007956	NM_001272234.1	0	+	1993222	2002540	0	12	65,467,186,94,49,58,1651,2824,149,1389,122,5497,	0,487,11341,25956,34632,44458,50599,55642,58614,58831,60292,60520,
**chr2L**	5305649	5310610	NM_001144320.2	0	+	5306024	5309720	0	3	517,348,2590,	0,1915,2371,
**chr2L**	5288943	5310996	NM_001298694.1	0	+	5307779	5309720	0	3	566,348,2976,	0,18621,19077,
**chr2L**	5305649	5311223	NM_001298695.1	0	+	5306024	5309720	0	3	517,348,3203,	0,1915,2371,
**chr2L**	18952205	18954431	NM_001299142.1	0	-	18952338	18953560	0	4	792,180,387,160,	0,952,1198,2066,
**chr2L**	18952205	18954431	NM_001299143.1	0	-	18952338	18953560	0	5	792,180,170,117,160,	0,952,1198,1468,2066,
**chr2L**	21237249	21258196	NM_057584.5	0	+	21253669	21257829	0	8	97,285,1759,291,150,103,155,451,	0,1031,16305,18288,18647,19696,19853,20496,
**chr2L**	21237249	21256186	NM_057585.3	0	+	21253669	21256067	0	5	97,285,1759,291,290,	0,1031,16305,18288,18647,
**chr2L**	5305649	5310996	NM_057843.4	0	+	5306024	5309720	0	3	517,348,2976,	0,1915,2371,
**chr2L**	3334874	3354829	NM_079909.3	0	-	3335656	3353392	0	9	978,267,157,639,256,412,210,1239,239,	0,1039,1367,1586,2288,2608,3352,17601,19716,
**chr2L**	21237249	21259675	NM_134296.3	0	+	21253669	21257829	0	8	171,285,1759,291,150,103,155,1930,	0,1031,16305,18288,18647,19696,19853,20496,

**chr2L**	3334874	3354829	NM_134924.2	0	-	3335656	3353392	0	9	978,267,157,639,256,412,210,1239,991,	0,1039,1367,1586,2288,2608,3352,17601,18964,
**chr2L**	7194907	7204264	NM_135259.3	0	-	7195555	7196674	0	5	733,350,124,545,326,	0,795,1204,1405,9031,
**chr2L**	9521209	9540060	NM_135449.4	0	+	9533581	9538654	0	9	231,181,238,1022,855,415,152,130,2256,	0,1953,12304,13677,14785,15702,16183,16403,16595,
**chr2L**	18952205	18954431	NM_136076.4	0	-	18952338	18953560	0	4	792,180,170,160,	0,952,1198,2066,
**chr2L**	5299646	5310625	NM_164639.2	0	+	5307779	5309720	0	3	105,348,2605,	0,7918,8374,
**chr2L**	7194907	7204264	NM_164741.2	0	-	7195555	7196674	0	6	733,350,124,383,107,330,	0,795,1204,1405,1843,9027,
**chr2L**	9521209	9540060	NM_164857.3	0	+	9533581	9538654	0	9	286,181,238,1022,855,415,152,130,2256,	0,1953,12304,13677,14785,15702,16183,16403,16595,
**chr2L**	21237249	21259675	NM_165364.2	0	+	21253669	21257829	0	8	155,285,1759,291,150,103,155,1930,	0,1031,16305,18288,18647,19696,19853,20496,
**chr2L**	3334874	3354829	NM_205900.2	0	-	3335656	3353392	0	9	978,267,157,639,256,412,210,1239,356,	0,1039,1367,1586,2288,2608,3352,17601,19599,
**chr2L**	9534747	9540060	NM_205945.2	0	+	9534899	9538654	0	7	46,1022,855,415,152,130,2256,	0,139,1247,2164,2645,2865,3057,
**chr2L**	9521209	9540045	NM_205946.2	0	+	9533581	9538654	0	8	286,238,1022,855,415,152,130,2241,	0,12304,13677,14785,15702,16183,16403,16595,
**chr2R**	10205453	10237348	NM_001103786.2	0	-	10208007	10237040	0	9	2675,151,244,147,104,164,169,228,351,	0,2737,2947,3252,3467,3647,3986,4426,31544,
**chr2R**	6087872	6131841	NM_001169590.2	0	-	6092587	6123560	0	6	5861,153,144,515,966,776,	0,5924,6137,6684,35009,43193,
**chr2R**	8600414	8612295	NM_001169618.2	0	+	8600581	8610888	0	6	815,99,210,345,393,2382,	0,7742,7897,8635,9040,9499,
**chr2R**	10203994	10227957	NM_001259307.3	0	-	10208007	10222606	0	10	4134,151,244,147,104,170,169,228,1548,629,	0,4196,4406,4711,4926,5106,5445,5885,18482,23334,
**chr2R**	10205453	10227957	NM_001273925.2	0	-	10207881	10222606	0	10	2675,151,244,147,104,170,169,228,1548,635,	0,2737,2947,3252,3467,3647,3986,4426,17023,21869,
**chr2R**	9177863	9179346	NM_001299308.1	0	-	9178433	9179219	0	3	681,144,517,	0,742,966,
**chr2R**	9177863	9181499	NM_001299309.1	0	-	9178433	9179219	0	4	681,144,396,63,	0,742,966,3573,
**chr2R**	23978800	23985177	NM_001299872.1	0	+	23980211	23985019	0	2	1945,1634,	0,4743,
**chr2R**	10203994	10234607	NM_001347757.1	0	-	10208007	10233311	0	9	4134,151,244,147,104,164,169,228,2584,	0,4196,4406,4711,4926,5106,5445,5885,28029,
**chr2R**	23979098	23985177	NM_057546.3	0	+	23980211	23985019	0	2	1647,1634,	0,4445,

**chr2R**	9177863	9181298	NM_080370.4	0	-	9178433	9181158	0	4	681,144,396,1550,	0,742,966,1885,
**chr2R**	23979894	23985177	NM_134290.1	0	+	23980211	23985019	0	2	851,1634,	0,3649,
**chr2R**	7446188	7451553	NM_136435.4	0	-	7446400	7449768	0	4	701,228,362,108,	0,765,3229,5257,
**chr2R**	11665767	11674782	NM_136849.2	0	+	11668578	11674431	0	8	50,259,935,151,126,786,286,539,	0,2654,3499,5262,6949,7277,8122,8476,
**chr2R**	24295398	24298111	NM_138062.3	0	+	24295649	24297991	0	3	451,1477,429,	0,509,2284,
**chr2R**	6090418	6169087	NM_165461.3	0	-	6092587	6152493	0	7	3315,153,144,515,167,482,332,	0,3378,3591,4138,61411,61650,78337,
**chr2R**	6091865	6157129	NM_165462.2	0	-	6092587	6152493	0	8	1868,153,144,515,167,482,211,200,	0,1931,2144,2691,59964,60203,64292,65064,
**chr2R**	6090418	6157129	NM_165463.2	0	-	6092587	6152493	0	8	3315,153,144,515,167,482,257,200,	0,3378,3591,4138,61411,61650,65739,66511,
**chr2R**	6090418	6131922	NM_165464.3	0	-	6092587	6131117	0	5	3315,153,144,515,857,	0,3378,3591,4138,40647,
**chr2R**	6092276	6131922	NM_165465.3	0	-	6092587	6123560	0	6	1457,153,144,515,966,857,	0,1520,1733,2280,30605,38789,
**chr2R**	8600414	8611238	NM_165611.3	0	+	8600581	8610888	0	5	815,210,345,393,1325,	0,7897,8635,9040,9499,
**chr2R**	9177863	9186342	NM_165658.2	0	-	9178433	9186133	0	4	681,144,396,227,	0,742,966,8252,
**chr2R**	9177863	9186342	NM_165659.2	0	-	9178433	9185732	0	4	681,144,396,1126,	0,742,966,7353,
**chr2R**	9177863	9186342	NM_165660.2	0	-	9178433	9185732	0	5	681,144,396,708,227,	0,742,966,7353,8252,
**chr2R**	9177863	9180338	NM_165663.3	0	-	9178433	9180267	0	4	681,144,396,590,	0,742,966,1885,
**chr2R**	11672055	11674782	NM_165845.2	0	+	11672214	11674431	0	5	243,126,786,286,539,	0,661,989,1834,2188,
**chr2R**	24294096	24298111	NM_166666.2	0	+	24294220	24297991	0	4	125,277,1477,429,	0,1476,1811,3586,
**chr2R**	10205453	10234607	NM_176121.3	0	-	10208007	10232780	0	9	2675,151,244,147,104,164,169,228,2584,	0,2737,2947,3252,3467,3647,3986,4426,26570,
**chr2R**	10205453	10227957	NM_176123.5	0	-	10208007	10222606	0	10	2675,151,244,147,104,170,169,228,1548,635,	0,2737,2947,3252,3467,3647,3986,4426,17023,21869,
**chr3L**	17558671	17576320	NM_001014590.2	0	-	17561769	17575831	0	4	4087,255,369,1528,	0,5934,6685,16121,
**chr3L**	17560157	17619325	NM_001259887.2	0	-	17561769	17598952	0	8	2601,255,369,508,282,613,709,675,	0,4448,5199,37302,38029,38663,42517,58493,
**chr3L**	17953357	18061453	NM_001259892.1	0	-	17953640	18059584	0	8	1692,1009,239,145,89,2038,129,508,	0,1857,6024,16005,39550,104879,107064,107588,
**chr3L**	17950952	18064696	NM_001259893.2	0	-	17953640	18059584	0	8	4097,1009,239,145,89,2038,433,186,	0,4262,8429,18410,41955,107284,109469,113558,
**chr3L**	21226608	21265613	NM_001259933.1	0	+	21227472	21264423	0	7	198,1134,233,142,641,276,1568,	0,649,4271,4560,36387,37103,37437,
**chr3L**	21226608	21265106	NM_001259934.1	0	+	21227472	21264423	0	7	54,1134,233,142,641,276,1061,	0,649,4271,4560,36387,37103

											,37437,
**chr3L**	15511888	15513202	NM_001274949.1	0	+	15512034	15513030	0	4	194,194,261,407,	0,283,587,907,
**chr3L**	15511051	15513202	NM_001274950.1	0	+	15511118	15513075	0	4	118,194,261,407,	0,1120,1424,<br>1744,
**chr3L**	17396030	17404619	NM_001300164.1	0	-	17396553	17403630	0	6	2300,137,146,232<br>,242,32,	0,2364,2556,<br>5580,7462,<br>8557,
**chr3L**	17953357	18026032	NM_001316451.1	0	-	17953640	18025415	0	6	1692,1009,239,145<br>,89,702,	0,1857,6024<br>,16005,39550,<br>71973,
**chr3L**	15511051	15513202	NM_079361.3	0	+	15511118	15513030	0	4	118,194,261,407,	0,1120,1424,<br>1744,
**chr3L**	17396030	17415811	NM_079401.3	0	-	17396553	17409863	0	6	2300,137,146,232,<br>648,591,	0,2364,2556,<br>5580,13194,<br>19190,
**chr3L**	17952161	17971439	NM_079409.3	0	-	17953640	17970909	0	5	2888,1009,239,<br>145,1796,	0,3053,7220,<br>17201,17482,
**chr3L**	21226918	21266503	NM_079471.3	0	+	21227472	21264423	0	6	1473,233,142,641<br>,276,2458,	0,3961,4250,<br>36077,36793,<br>37127,
**chr3L**	3055907	3070839	NM_139508.3	0	-	3056724	3070539	0	8	845,104,278,153,<br>124,115,59,678,	0,911,1085,1522,<br>1733,1921,<br>2158,14254,
**chr3L**	15511051	15513202	NM_168622.2	0	+	15511118	15513030	0	4	118,182,261,407,	0,1120,1424,<br>1744,
**chr3L**	15511051	15513202	NM_168623.2	0	+	15511118	15512369	0	3	118,565,407,	0,1120,1744,
**chr3L**	17560157	17619325	NM_168739.3	0	-	17561769	17599024	0	8	2601,255,369,508,<br>282,613,709,675,	0,4448,5199,<br>37302,38029,<br>38663,42517,<br>58493,
**chr3L**	17560157	17619325	NM_168740.3	0	-	17561769	17598907	0	8	2601,255,369,508,<br>282,613,709,675,	0,4448,5199,<br>37302,38029,<br>38663,42517,<br>58493,
**chr3L**	17560157	17576626	NM_168741.2	0	-	17561769	17575831	0	4	2601,255,369,1834,	0,4448,5199,<br>14635,
**chr3L**	17953357	18059598	NM_168755.1	0	-	17953640	18059584	0	6	1692,1009,239,145,<br>89,1362,	0,1857,6024,<br>16005,39550,<br>104879,
**chr3L**	17952161	17999821	NM_168756.2	0	-	17953640	17999131	0	6	2888,1009,239,145,<br>89,1399,	0,3053,7220,<br>17201,40746,<br>46261,
**chr3L**	17953357	17961814	NM_168757.1	0	-	17953640	17961068	0	4	1692,1009,239,810,	0,1857,6024,<br>7647,
**chr3L**	21240346	21266503	NM_168892.3	0	+	21263111	21264423	0	4	457,641,276,2458,	0,22649,23365,<br>23699,
**chr3L**	17396030	17421514	NM_170636.2	0	-	17396553	17409863	0	6	2300,137,146,232,<br>648,161,	0,2364,2556,<br>5580,13194,<br>25323,
**chr3L**	17396030	17406521	NM_170637.2	0	-	17396553	17406427	0	5	2300,137,146,<br>232,112,	0,2364,2556,<br>5580<br>,10379,
**chr3L**	17396030	17403734	NM_170638.2	0	-	17396553	17403630	0	5	2300,137,146,<br>232,242,	0,2364,2556,<br>5580,<br>7462,
**chr3L**	15511562	15513202	NM_206371.3	0	+	15511602	15513030	0	4	70,194,261,407,	0,609,913,<br>1233,
**chr3L**	3249339	3252400	NR_048218.2	0	+	3252400	3252400	0	1	3061,	0,
**chr3L**	3249589	3254504	NR_124769.1	0	+	3254504	3254504	0	1	4915,	0,
**chr3L**	3250593	3251002	NR_133165.1	0	+	3251002	3251002	0	1	409,	0,
**chr3R**	6389161	6405895	NM_001144543.3	0	-	6389558	6392007	0	10	524,176,114,105,121,<br>506,65,1438,180,518,	0,596,849,<br>1040,<br>1227,1426,<br>1999,<br>2143,4570,<br>16216,
**chr3R**	6389161	6405895	NM_001144544.3	0	-	6389558	6391593	0	12	524,176,114,105,121,<br>506,65,684,80,180,<br>283,154,	0,596,849,<br>1040,<br>1227,1426,

											1999, 2143,3718, 4570, 16216, 16580,
**chr3R**	6389161	6405895	NM_001170060.2	0	-	6389558	6393873	0	11	524,176,114,105,121, 506,65,684,180,106, 347,	0,596,849, 1040, 1227,1426, 1999, 2143,4570, 16216, 16387,
**chr3R**	21051382	21060653	NM_001170197.1	0	-	21052187	21058467	0	11	860,71,92,116,446, 159,156,139,165,185, 2393,	0,3962,4249, 4404 ,4581,5085, 5312, 6193,6404, 6633, 6878,
**chr3R**	21053203	21060653	NM_001260292.1	0	-	21053530	21058467	0	12	415,71,92,116,446, 159,156,139,165, 185,208,69,	0,2141,2428, 2583, 2760,3264, 3491, 4372,4583, 4812, 5057,7381,
**chr3R**	21051382	21060800	NM_001275866.1	0	-	21052058	21058467	0	12	860,71,92,116,446, 159,156,139,165, 185,208,56,	0,3962,4249, 4404 ,4581,5085, 5312, 6193,6404, 6633, 6878,9362,
**chr3R**	31384398	31391893	NM_001276210.1	0	+	31387110	31390659	0	9	101,323,247,74,146 ,215,230,253,1305,	0,2677,3535, 4638, 4770,4982, 5260, 5547,6190,
**chr3R**	31382108	31391893	NM_001276211.1	0	+	31387110	31390659	0	9	227,323,247,74,146 ,215,230,253,1305,	0,4967,5825, 6928, 7060,7272, 7550, 7837,8480,
**chr3R**	5257036	5269046	NM_001300242.1	0	+	5257564	5267840	0	5	1085,280,81,140, 1585,	0,9716,10084, 10226,10425,
**chr3R**	21051561	21060653	NM_079701.5	0	-	21052187	21058467	0	12	681,71,92,116,446, 159,156,139,165, 185,208,372,	0,3783, 4070, 4225,4402, 4906, 5133,6014, 6225, 6454,6699, 8720,
**chr3R**	4781119	4784722	NM_141238.2	0	-	4781270	4784684	0	4	846,1809,148,290,	0,1167,3107, 3313,
**chr3R**	5264938	5268598	NM_141268.3	0	+	5265539	5267840	0	5	699,280,81,140,1137,	0,1814,2182, 2324, 2523,
**chr3R**	6389161	6405895	NM_141397.5	0	-	6389558	6392007	0	11	524,176,114,105, 121,506,65,1438, 80,180,154,	0,596,849, 1040, 1227,1426, 1999 ,2143,3718, 4570 ,16580,
**chr3R**	26461526	26470008	NM_143224.4	0	+	26466681	26469402	0	7	120,486,215,544, 653,147,725,	0,5058,5605, 6017 ,6619,7330, 7757,
**chr3R**	28878023	28880122	NM_143387.2	0	-	28878080	28880096	0	1	2099,	0,
**chr3R**	31382108	31391893	NM_143608.2	0	+	31387110	31390591	0	10	227,323,247,74, 146,215,230,253, 62,1305,	0,4967,5825, 6928, 7060,7272, 7550,

											7837,8189, 8480,
**chr3R**	5257036	5268371	NM_169036.2	0	+	5257564	5267840	0	5	1085,280,81,140,910,	0,9716,10084, 10226,10425,
**chr3R**	5264938	5269507	NM_169037.3	0	+	5266795	5267840	0	6	264,280,81,140, 416,426,	0,1814,2182, 2324,2523, 4143,
**chr3R**	6389161	6405895	NM_169141.4	0	-	6389558	6392007	0	11	524,176,114,105, 121,506,65,1438 ,80,180,518,	0,596,849, 1040 ,1227,1426, 1999, 2143,3718, 4570, 16216,
**chr3R**	21051382	21060800	NM_169944.3	0	-	21052187	21058467	0	12	860,71,92,116,446 ,159,156,139,165 ,185,208,56,	0,3962,4249, 4404 ,4581,5085, 5312, 6193,6404, 6633, 6878,9362,
**chr3R**	26465539	26470008	NM_170273.2	0	+	26466681	26469402	0	7	112,486,215,544,653 ,147,725,	0,1045,1592, 2004 ,2606,3317, 3744,
**chr3R**	31384398	31391893	NM_170548.2	0	+	31387110	31390591	0	10	101,323,247,74, 146,215,230,253 ,62,1305,	0,2677,3535, 4638 ,4770,4982, 5260 ,5547,5899, 6190,
**chr3R**	21054647	21060653	NM_206529.2	0	-	21054959	21058467	0	12	394,71,92,116, 446,159,156, 139,165,185,208 ,372,	0,697,984, 1139, 1316,1820, 2047 ,2928,3139, 3368 ,3613,5634,

TableS2. Top9 20E-dependent transcripts where NELF-B promoter-bound level decrease upon NELFA- RNAi more than 2 times

Chrom	Start	End	Name	Score	Strand	ThickStart	ThickEnd	ItemRGB	BlockCount	BlockSizes	BlockStarts
**chr3R**	31384398	31391893	NM_170548.2	0	+	31387110	31390591	0	10	101,323,247,74, 146,215,230,253, 62,1305,	0,2677,3535,4638, 4770,4982,5260, 5547,5899,6190,
**chr2R**	9177863	9186342	NM_165658.2	0	-	9178433	9186133	0	4	681,144,396,227,	0,742,966,8252,
**chr3L**	21240346	21266503	NM_168892.3	0	+	21263111	21264423	0	4	457,641,276,2458,	0,22649,23365, 23699,
**chr2R**	10203994	10227957	NM_001259307.3	0	-	10208007	10222606	0	10	4134,151,244,147, 104,170,169,228, 1548,629,	0,4196,4406,4711, 4926,5106,5445, 5885,18482,23334,
**chr3R**	28878023	28880122	NM_143387.2	0	-	28878080	28880096	0	1	2099,	0,
**chr2R**	24294096	24298111	NM_166666.2	0	+	24294220	24297991	0	4	125,277,1477,429,	0,1476,1811,3586,
**chrX**	1622731	1645162	NM_001201597.1	0	+	1624521	1643765	0	8	257,570,189,454, 274,140,51,2278,	0,1406,13100,13364, 13907,14268,14552, 20153,
**chr2L**	3334874	3354829	NM_079909.3	0	-	3335656	3353392	0	9	978,267,157,639, 256,412,210,1239, 239,	0,1039,1367,1586, 2288,2608,3352, 17601,19716,
**chr2L**	7194907	7204264	NM_164741.2	0	-	7195555	7196674	0	6	733,350,124,383, 107,330,	0,795,1204,1405, 1843,9027,

TableS3. 27 20E-dependent transcripts where NELF-B promoter-bound level remains unchanged upon NELFA-B RNAi

Chrom	Start	End	Name	Score	Strand	ThickStart	ThickEnd	ItemRGB	BlockCount	BlockSizes	BlockStarts
**chr2R**	8600414	8611238	NM_165611.3	0	+	8600581	8610888	0	5	815,210,345,393, 1325,	0,7897,8635 ,9040,9499,
**chr3R**	6389161	6405895	NM_001144544.3	0	-	6389558	6391593	0	12	524,176,114,105, 121,506,65,684,80, 180,283,154,	0,596,849,1040, 1227,1426,1999, 2143,3718,4570, 16216,16580,
**chr3R**	5264938	5268598	NM_141268.3	0	+	5265539	5267840	0	5	699,280,81,140, 1137,	0,1814,2182, 2324,2523,
**chr2L**	9521209	9540060	NM_164857.3	0	+	9533581	9538654	0	9	286,181,238,1022 ,855,415,152,130, 2256,	0,1953,12304, 13677,14785, 15702,16183, 16403,16595,
**chr2L**	21237249	21259675	NM_165364.2	0	+	21253669	21257829	0	8	155,285,1759,291, 150,103,155,1930,	0,1031,16305, 18288,18647, 19696,19853, 20496,
**chr2L**	5305649	5310996	NM_057843.4	0	+	5306024	5309720	0	3	517,348,2976,	0,1915,2371,
**chr3L**	17560157	17576626	NM_168741.2	0	-	17561769	17575831	0	4	2601,255,369, 1834,	0,4448,5199, 14635,
**chr3L**	17396030	17421514	NM_170636.2	0	-	17396553	17409863	0	6	2300,137,146,232 ,648,161,	0,2364,2556, 5580,13194, 25323,
**chr3L**	15511051	15513202	NM_168623.2	0	+	15511118	15512369	0	3	118,565,407,	0,1120,1744,
**chr3R**	26461526	26470008	NM_143224.4	0	+	26466681	26469402	0	7	120,486,215,544 ,653,147,725,	0,5058,5605 ,6017,6619, 7330,7757,
**chr2L**	18952205	18954431	NM_001299142.1	0	-	18952338	18953560	0	4	792,180,387,160,	0,952,1198, 2066,
**chr3L**	5654680	5657384	NM_139726.2	0	+	5654979	5657119	0	5	366,137,149,151, 349,	0,1638,1924, 2142,2355,
**chrX**	1941939	2005462	NM_001272233.2	0	+	1993222	2002540	0	11	954,186,94,49,58, 1175,2824,149, 1389,122,3003,	0,11341,25956, 34632,44458, 51075,55642, 58614,58831, 60292,60520,
**chr3L**	17952161	17999821	NM_168756.2	0	-	17953640	17999131	0	6	2888,1009,239, 145,89,1399,	0,3053,7220 ,17201,40746 ,46261,
**chr2R**	11665767	11674782	NM_136849.2	0	+	11668578	11674431	0	8	50,259,935,151, 126,786,286,539,	0,2654,3499, 5262,6949, 7277,8122,8476,
**chr3L**	17953357	17961814	NM_168757.1	0	-	17953640	17961068	0	4	1692,1009,239,810,	0,1857,6024, 7647,
**chr2R**	8611378	8614155	NM_136552.3	0	-	8611893	8614155	0	2	681,1122,	0,1655,
**chr3L**	3055907	3070839	NM_139508.3	0	-	3056724	3070539	0	8	845,104,278,153, 124,115,59,678,	0,911,1085, 1522,1733, 1921,2158, 14254,
**chr2R**	7446188	7451553	NM_136435.4	0	-	7446400	7449768	0	4	701,228,362,108,	0,765,3229, 5257,
**chr2R**	23979098	23985177	NM_057546.3	0	+	23980211	23985019	0	2	1647,1634,	0,4445,
**chr2L**	14844992	14851749	NM_001014484.2	0	-	14845116	14849122	0	7	369,277,742,250, 304,103,468,	0,424,760, 3012,3681, 4046,6289,
**chr3R**	21051561	21060653	NM_079701.5	0	-	21052187	21058467	0	12	681,71,92,116,446, 159,156,139,165, 185,208,372,	0,3783,4070 ,4225,4402, 4906,5133, 6014,6225, 6454,6699,8720,
**chr3L**	13860026	13866905	NM_140408.3	0	-	13860756	13866691	0	4	1772,235,119, 303,	0,1892,5290 ,6576,
**chr2R**	6092276	6131922	NM_165465.3	0	-	6092587	6123560	0	6	1457,153,144, 515,966,857,	0,1520,1733, 2280,30605, 38789,
**chr3R**	30243977	30246703	NM_143527.3	0	-	30244105	30246621	0	3	723,601,143,	0,1546,2583,

**chr3R**	4781119	4784722	NM_141238.2	0	-	4781270	4784684	0	4	846,1809,148,290,	0,1167,3107, 3313,

Table. S4. List of 20E-dependent transcripts induced both in embryo and prepupa

Chrom	Start	End	Name	Score	Strand	ThickStart	ThickEnd	ItemRGB	BlockCount	BlockSizes	BlockStarts
**chr2R**	10203994	10227957	NM_001259307.3	0	-	10208007	10222606	0	10	4134,151,244,147, 104,170,169,228, 1548,629,	0,4196,4406, 4711,4926 ,5106,5445, 5885,18482, 23334,
**chrX**	1941939	2005462	NM_001272233.2	0	+	1993222	2002540	0	11	954,186,94,49,58, 1175,2824,149, 1389,122,3003,	0,11341,25956 ,34632,44458 ,51075,55642, 58614,58831, 60292,60520,
**chr2L**	3334874	3354829	NM_079909.3	0	-	3335656	3353392	0	9	978,267,157,639, 256,412,210, 1239,239,	0,1039,1367, 1586,2288, 2608,3352, 17601,19716,
**chr2L**	5305649	5310996	NM_057843.4	0	+	5306024	5309720	0	3	517,348,2976,	0,1915,2371,
**chr2L**	21237249	21259675	NM_165364.2	0	+	21253669	21257829	0	8	155,285,1759,291, 150,103,155,1930,	0,1031,16305, 18288,18647, 19696,19853 ,20496,
**chr3L**	17952161	17999821	NM_168756.2	0	-	17953640	17999131	0	6	2888,1009,239, 145,89,1399,	0,3053,7220, 17201,40746 ,46261,
**chr3L**	17953357	17961814	NM_168757.1	0	-	17953640	17961068	0	4	1692,1009,239,810,	0,1857,6024, 7647,
**chr2R**	6092276	6131922	NM_165465.3	0	-	6092587	6123560	0	6	1457,153,144, 515,966,857,	0,1520,1733, 2280,30605, 38789,
**chr2R**	8600414	8611238	NM_165611.3	0	+	8600581	8610888	0	5	815,210,345, 393,1325,	0,7897,8635 ,9040,9499,
**chr3L**	3055907	3070839	NM_139508.3	0	-	3056724	3070539	0	8	845,104,278, 153,124,115 ,59,678,	0,911,1085, 1522,1733, 1921,2158, 14254,
**chr3R**	28878023	28880122	NM_143387.2	0	-	28878080	28880096	0	1	2099,	0,
**chr2R**	23979098	23985177	NM_057546.3	0	+	23980211	23985019	0	2	1647,1634,	0,4445,
